# A comparison of behavioral paradigms assessing spatial memory in tree shrews

**DOI:** 10.1093/cercor/bhad283

**Published:** 2023-08-26

**Authors:** Cheng-Ji Li, Yi-Qing Hui, Rong Zhang, Hai-Yang Zhou, Xing Cai, Li Lu

**Affiliations:** Key Laboratory of Animal Models and Human Disease Mechanisms of Yunnan Province, Kunming Institute of Zoology, Chinese Academy of Sciences, Kunming, Yunnan 650201, China; National Research Facility for Phenotypic & Genetic Analysis of Model Animals (Primate Facility), Kunming Institute of Zoology, Chinese Academy of Sciences, Kunming, Yunnan 650107, China; Key Laboratory of Animal Models and Human Disease Mechanisms of Yunnan Province, Kunming Institute of Zoology, Chinese Academy of Sciences, Kunming, Yunnan 650201, China; Key Laboratory of Animal Models and Human Disease Mechanisms of Yunnan Province, Kunming Institute of Zoology, Chinese Academy of Sciences, Kunming, Yunnan 650201, China; National Research Facility for Phenotypic & Genetic Analysis of Model Animals (Primate Facility), Kunming Institute of Zoology, Chinese Academy of Sciences, Kunming, Yunnan 650107, China; Key Laboratory of Animal Models and Human Disease Mechanisms of Yunnan Province, Kunming Institute of Zoology, Chinese Academy of Sciences, Kunming, Yunnan 650201, China; Key Laboratory of Animal Models and Human Disease Mechanisms of Yunnan Province, Kunming Institute of Zoology, Chinese Academy of Sciences, Kunming, Yunnan 650201, China; National Research Facility for Phenotypic & Genetic Analysis of Model Animals (Primate Facility), Kunming Institute of Zoology, Chinese Academy of Sciences, Kunming, Yunnan 650107, China; Key Laboratory of Animal Models and Human Disease Mechanisms of Yunnan Province, Kunming Institute of Zoology, Chinese Academy of Sciences, Kunming, Yunnan 650201, China; National Research Facility for Phenotypic & Genetic Analysis of Model Animals (Primate Facility), Kunming Institute of Zoology, Chinese Academy of Sciences, Kunming, Yunnan 650107, China; Center for Excellence in Brain Science and Intelligence Technology, Chinese Academy of Sciences, Shanghai 200031, China

**Keywords:** tree shrews, spatial memory, behavioral paradigm, cheeseboard maze, hippocampal lesion

## Abstract

Impairments in spatial navigation in humans can be preclinical signs of Alzheimer's disease. Therefore, cognitive tests that monitor deficits in spatial memory play a crucial role in evaluating animal models with early stage Alzheimer's disease. While Chinese tree shrews (*Tupaia belangeri*) possess many features suitable for Alzheimer's disease modeling, behavioral tests for assessing spatial cognition in this species are lacking. Here, we established reward-based paradigms using the radial-arm maze and cheeseboard maze for tree shrews, and tested spatial memory in a group of 12 adult males in both tasks, along with a control water maze test, before and after bilateral lesions to the hippocampus, the brain region essential for spatial navigation. Tree shrews memorized target positions during training, and task performance improved gradually until reaching a plateau in all 3 mazes. However, spatial learning was compromised post-lesion in the 2 newly developed tasks, whereas memory retrieval was impaired in the water maze task. These results indicate that the cheeseboard task effectively detects impairments in spatial memory and holds potential for monitoring progressive cognitive decline in aged or genetically modified tree shrews that develop Alzheimer's disease-like symptoms. This study may facilitate the utilization of tree shrew models in Alzheimer's disease research.

## Introduction

Environmental navigation is crucial for daily life in animals. Lesions to the entorhinal-hippocampal circuit compromise an animal's spatial-learning ability (Squire 1992; Steffenach et al. 2005). In humans, damage to the entorhinal-hippocampal circuit is primarily observed in patients suffering from Alzheimer's disease (AD), an age-related progressive neurodegenerative disorder (Braak and Braak 1991; Adams et al. 2022). Although loss of episodic memory is a standard clinical diagnostic measure for AD (Dubois et al. 2014), deficits in spatial navigation are more sensitive at identifying at-risk individuals (Coughlan et al. 2018). Emerging data obtained from rodent models have also revealed early AD pathophysiology in the entorhinal-hippocampal circuit, suggesting that navigational deficits may be a more sensitive cognitive marker for this disease (Fu et al. 2017; Jun et al. 2020; Ying et al. 2022). Thus, spatial navigation abilities are the most vulnerable to damage in the entorhinal-hippocampal circuit and may serve as a cognitive marker for incipient AD, both in human patients and animal models.

The Chinese tree shrew (*Tupaia belangeri*) is a squirrel-like mammal in the order Scandentia (Zheng et al. 2014). Because of its short reproductive cycle and phylogenetic affinity to primates (Xu et al. 2012; Fan et al. 2013, 2019; Ye et al. 2021), tree shrews have been used as experimental model alternatives to primates in a variety of human diseases, including infections, cancer (Cao et al. 2003; Xiao et al. 2017; Yao 2017; Li et al. 2018), and neuropsychiatric disorders (Fuchs 2005; Zambello et al. 2010; Pryce and Fuchs 2017; Ni et al. 2020; Savier et al. 2021). The tree shrew beta-amyloid (Aβ) amino acid sequence is identical to that in humans (Pawlik et al. 1999), and the expression patterns of AD-related genes are similar (Fan et al. 2018; Ye et al. 2021). Additionally, Aβ deposition and tau over-phosphorylation, 2 key molecular markers of AD, have also been observed in the brains of aged tree shrews (Yamashita et al. 2010, 2012; Fan et al. 2018), suggesting that AD may occur naturally in those animals, similar to nonhuman primates (Paspalas et al. 2018). Moreover, studies have shown that intracerebroventricular injections of Aβ fragments into the tree shrew brain can result in profound molecular, cellular, and cognitive changes (Lin et al. 2016; Wang et al. 2020), which closely resemble human AD pathology (Masters et al. 2015). Collectively, these pioneer studies suggest that tree shrews may serve as promising animal models for future AD research.

Cognitive impairment serves as a crucial determinant in establishing the validity of an AD model (Chen and Zhang 2022). Several behavioral paradigms have been developed to study cognitive functions in tree shrews, such as associative learning (Ohl et al. 1998), object recognition (Khani and Rainer 2012; Nair et al. 2014), contextual fear conditioning (Shang et al. 2015), and visual discrimination (Mustafar et al. 2018; Li et al. 2022). However, these tasks are not specifically designed to evaluate memory of spatial location, an important measure of spatial cognition. Although tree shrews have been tested in water mazes for loss of spatial memory (Wang et al. 2020), this paradigm is only sensitive for animals with severe hippocampal damage (Moser et al. 1993, 1995). Thus, taken together, these behavioral tests are not suitable for detecting mild spatial cognitive impairment representing the early stages of AD.

In the current study, we aimed to establish robust behavioral paradigms to detect small changes in spatial memory in tree shrews, thus allowing reliable monitoring of progressive spatial cognitive decline in animals developing AD. We established tasks for tree shrews using radial-arm and cheeseboard mazes, and compared their sensitivity to spatial memory loss in hippocampal-lesioned animals using the pre-established water maze test.

## Materials and methods

### Subjects

**Fig. 1:**
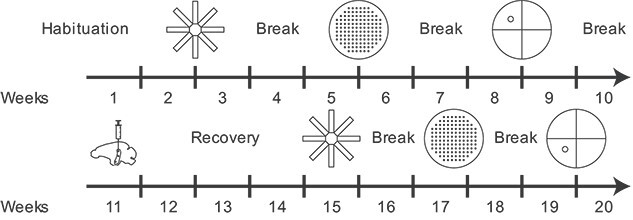
Experimental design and timeline.

All experiments were conducted at the Kunming Institute of Zoology (Kunming, China), in accordance with the guidelines for the care and use of laboratory animals and with approval by the Institutional Animal Care and Use Committee of Kunming Institute of Zoology (KIZ-IACUC-TE-2022-02-001). Fifteen adult male Chinese tree shrews (*T. belangeri*), aged 13–15 months and weighing 110–150 g at the start of the experiment, were initially included in the study (TS094–TS108). The selected animals had no previous exposure to behavioral tests and were individually housed in wire cages (44 × 38 × 35 cm), equipped with a dark nest box (36 × 16 × 20 cm) under standard laboratory conditions (temperature: 20–26°C, relative humidity: 40–60%). The tree shrews were maintained on a 12-h light/dark schedule, with all testing procedures conducted in the light phase. Three animals were removed before testing started because of health issues (TS098 and TS107, vulnerable to food restriction) or test aversion (TS105, untrainable). The remaining 12 animals underwent behavioral testing using the radial-arm, cheeseboard, and water mazes sequentially, with a 1-week interval between tasks (Figs. 1 and S1). The tree shrews were mildly food restricted during the testing period in the radial-arm and cheeseboard mazes (maintained at 85–90% of free-feeding body weight), otherwise they were fed *ad libitum* (Supplementary Fig. S2a). The 12 tree shrews received neurotoxic lesions to the hippocampus after the first round of behavioral tests, with 11 later tested on the same tasks. TS104 died during surgery, possibly because of isoflurane overdose, and was excluded from pre- and post-lesion comparison. Running speeds were slightly higher in the cheeseboard maze after bilateral lesions to the hippocampus (Supplementary Fig. S2b), whereas swimming speeds in the water maze were not altered (Supplementary Fig. S2c).

### Experimental design

The experimental design, shown in Fig. 1, involved the sequential training and testing of the 12 tree shrews using the radial-arm, cheeseboard, and water maze behavioral paradigms, specifically designed to assess spatial memory. The learning profiles for each behavioral test were obtained by testing the animals consecutively until their learning curves plateaued. After this, the animals were subjected to neurotoxic procedures to induce bilateral hippocampal lesions to impair spatial memory ability. After a recovery period, the same tree shrews were retested in the cognitive tasks to measure spatial memory loss. This experimental design allowed each animal to serve as its own control, thereby minimizing interindividual variability, and enabled comparison of behavioral paradigms within the same animal. Analysis of variance (ANOVA) for repeated measures and tests for paired samples were used to compare spatial learning before and after the lesions. Experimental details are described in the following sections.

### Behavioral apparatus

#### Radial-arm maze

The radial-arm maze was originally designed to assess working memory and reference (spatial) memory in rats (Olton and Samuelson 1976). Here, the maze (128 cm in diameter and 28 cm in height) was constructed by SANS Biological Technology using white plastic (floor, doors, lower 1/3 of walls) and transparent plexiglass (upper 2/3 of walls and lids; Supplementary Fig. S1a). The maze was positioned in a well-lit room (3.0 × 2.5 m) and placed on a platform (PF) 36 cm above the ground, surrounded by numerous visual cues. The maze comprised a center region (28 cm in diameter) and 8 equally spaced arms (50 cm in length and 10 cm in width). A small cake reward (made of water, wheat flour, egg, pumpkin, sugar, vegetable oil, starch, milk powder, glucose syrup, salt, and food additives. Nutrition facts (per 100 g): energy 1,458 kJ; protein 8.6 g; fat 11.8 g; carbohydrate 51.5 g; sodium 286 mg) was placed in a square bowl (9.0 × 9.0 × 5.2 cm) positioned at the end of each arm, not visible to the animal at the other end of the arm. Odor cues were obscured by large pieces of cake placed near the end of each arm outside the maze. Prior to testing, the animals were pretrained to enter all 8 arms to receive cake rewards. During testing, the 8 arms were separated into 1 closed start arm (arm 5), 3 baited open arms (arms 2, 4, and 7 before lesion; arms 3, 6, and 8 after lesion), and 4 unbaited open arms. At the start of each test day, each animal was transferred to the start arm and allowed to acclimate for several minutes. The start arm was covered by a towel during reward placement to ensure the animal waiting inside had no hint of the reward locations. For each trial, the tree shrew walked into the center region after the start arm was opened (start signal) and entered the remaining 7 arms to find rewards (Supplementary Fig. S1b). The trial ended when all 3 rewards had been consumed or after 2 min. Once the start arm was reopened (stop signal), the animal returned to the start arm (manually guided if necessary) to receive a fourth cake reward and waited 1 min for the next trial to begin. Testing for each animal concluded at 25 trials or 50 min, whichever occurred first. Urine marks were removed with bleach after each trial, and the entire maze was cleaned thoroughly with 70% alcohol once the testing for each animal was completed.

#### Cheeseboard maze

The cheeseboard maze was originally designed for navigation studies in rats (Dupret et al. 2010). Here, the maze was constructed with steel walls and a white plastic floor, measuring 150 cm in diameter and 50 cm in height. The maze was placed on a PF 36 cm above the floor in a well-lit room (3.0 × 2.5 m) decorated with numerous visual cues to facilitate animal orientation. The maze floor was divided into 4 quadrants, each containing 30 equally spaced (10 cm) wells (3.0 cm in diameter, 3.0 cm in depth). A transparent start box (22 × 16 × 20 cm) was positioned at the boundary of quadrants 3 and 4 near the wall (Supplementary Fig. S1c). Prior to testing, the animals were pretrained to navigate in the maze and consume cake rewards placed in 3 distant wells. During the test, 3 cake rewards were placed in wells that met the following criteria: (i) the wells were not selected on the previous test day, (ii) the distance between any 2 wells was >30 cm, and (iii) the area of the triangle formed by the 3 reward wells was 11–12 dm^2^. The locations of reward wells before lesions were: test day 1: [−3, 4; −1, −3; 3, −1]; test day 2: [2, 5; −3, 5; −1, −2]; test day 3: [4, 1; −2, 5; −4, 2]; test day 4: [3, 1; −4, 3; −4, −2]; test day 5: [3, −1; −1, 4; −4, −1]; test day 6: [3, 4; −4, 3; −2, −2]. The locations of reward wells after hippocampal lesions were: test day 1: [3, 4; 1, −3; −3, −1]; test day 2: [−2, 5; 3, 5; 1, −2]; test day 3: [−4, 1; 2, 5; 4, 2]; test day 4: [−3, 1; 4, 3; 4, −2]; test day 5: [−3, −1; 1, 4; 4, −1]. The spatial configurations of reward wells before and after lesions were symmetrical with respect to the vertical midline. To prevent the tree shrews from relying on local cues to solve the task, rewards were not visible to the animal, and odor cues were obscured using a layer of cake crumbs evenly spread beneath the floor. At the start of each test day, each animal was transferred to the start box and allowed to acclimate for several minutes. The start box was covered by a towel during reward placement to ensure the animal waiting inside had no hint of the reward locations. During each trial, after the start box was opened (start signal), the tree shrew was free to navigate the maze to locate and consume 3 cake rewards (Supplementary Fig. S1d). The trial ended when all 3 rewards had been consumed or after 2 min. Once the start box was reopened (stop signal), the animal returned to the start box (manually guided if necessary) to receive a fourth cake reward and waited 1 min for the next trial to begin. Testing for each animal concluded at 25 trials or 50 min, whichever occurred first. Urine marks were removed with bleach after each trial, and the entire maze was cleaned thoroughly with 70% alcohol once the testing for each animal was completed.

#### Water maze

The water maze was originally designed to assess spatial memory in rats (Morris 1984). Here, the steel swimming pool (150 cm in diameter and 50 cm in depth) was constructed with a featureless, black inner surface (SANS Biological Technology, China). The maze was positioned in a well-lit room (3.0 × 2.5 m) with numerous visual cues. The pool was filled to a depth of 20 cm with water maintained at a temperature of 18°C, to which 60 g of titanium dioxide powder was added. A white metal PF measuring 14 cm in diameter was submerged 1.5 cm below the water surface in the center of quadrant 2 (before lesion) or quadrant 3 (after lesion) during training. During pretraining (day 0), the water level was lowered 3 cm to make the PF visible to the animal (Supplementary Fig. S1e). Subsequently (days 1–7), the tree shrews were trained for 4 trials per day at 30-min intervals. Each trial was begun by releasing the animal into the water with its face oriented toward the pool wall in 1 of the 4 quadrants selected at random. If the tree shrew failed to locate the PF within 60 s, it was manually guided onto it. The animal was allowed to stay on the PF for 20 s. During the spatial probe test, the tree shrew was released from quadrant 4 (before lesion) or quadrant 1 (after lesion), with swimming tracked for a duration of 60 s. Upon the completion of each trial or test, the animal was dried with a towel and allowed to rest in its nest box beside a heater.

### Behavioral data collection

Animal behavior during training and testing was monitored by an overhead camera mounted 1.9 m above the mazes. Videos were captured at a resolution of 640 × 480 pixels and sampling rate of 50 Hz in.avi format using open-source Captura software (v9.0.0). The tree shrew movements were then tracked off-line using open-source DeepLabCut software (RRID: SCR_021391), which employs transfer learning with deep neural networks for markerless pose estimation (Mathis et al. 2018). To train the network model for a given animal, 600–1,000 frames from 4 to 7 videos were manually labeled to identify the snout, eyes, and tail base, when necessary, with training performed 900,000 times to ensure accuracy. The trained model was then used to automatically label all videos for the animal. Any errors identified in DeepLabCut outputs (time-position series of labeled points), such as discontinuities and sudden jumps, were manually corrected using custom MATLAB script (RRID: SCR_001622).

### Behavioral analyses

The performance of the tree shrews in the behavioral tests was assessed using custom MATLAB script, designed to analyze data obtained from DeepLabCut. To ensure result accuracy, the MATLAB output data were verified by manual inspection of the original trial videos.

#### Radial-arm maze

Snout positions of the tree shrews were used to analyze task performance. A valid arm visit was defined as a snout position > 35 cm into the arm.

Working memory error rate for each trial was determined as:


$$ \mathrm{Working}\ \mathrm{memory}\ \mathrm{error}\ \mathrm{rate}=\frac{\mathrm{No}.\mathrm{repeated}\ \mathrm{entries}\ \mathrm{into}\ \mathrm{all}\ 7\ \mathrm{arms}}{\mathrm{No}.\mathrm{entries}\ \mathrm{into}\ \mathrm{all}\ 7\ \mathrm{arms}} $$

Reference (spatial) memory error rate for each trial was determined as: 


$$ \mathrm{Reference}\ \mathrm{memory}\ \mathrm{error}\ \mathrm{rate}=\frac{\mathrm{No}.\mathrm{unbaited}\ \mathrm{arms}\ \mathrm{entered}}{\mathrm{No}.\mathrm{arms}\ \mathrm{for}\ \mathrm{selection}\ (7)} $$

In instances where the animal was unable to locate all 3 rewards before the end of the trial, only arm entries within the first 2 min were included for analysis. A trial was deemed correct if all 3 rewards were found without erroneous arm visits.

#### Cheeseboard maze

Both snout and head (midpoint of left and right eyes) positions of the tree shrews were used as tracked variables. A valid visit to a reward well was defined as a snout position in the well longer than 0.1 s. A trial was deemed successful if all 3 rewards were found within 2 min. Only trials that occurred after the first successful trial on each test day were included in subsequent analyses.

Route scores were used to evaluate spatial memory of reward locations:


$$ \mathrm{Routescore}=\frac{\mathrm{D}1+\mathrm{D}2+\mathrm{D}3\ }{\mathrm{Travel}\ \mathrm{length}\ \mathrm{from}\ \mathrm{start}\ \mathrm{box}\ \mathrm{to}\ 3\ \mathrm{reward}\ \mathrm{wells}} $$

where D1 is the distance between the start box and first reward well visited, D2 is the distance between the first and second reward wells, and D3 is the distance between the second and third reward wells. If a trial was unsuccessful, the route score was defined as 0. Test days on which the animal performed 5 or fewer successful trials were considered unmotivated and were excluded from analyses.

#### Water maze

Head positions of the tree shrews were used to assess water maze performance. Escape latency and distance were calculated as the time and travel length, respectively, that the animal took before climbing onto the PF. A trial was deemed successful if the animal found the PF. Escape latency was defined as 60 s in unsuccessful trials, and escape distance was defined as length traveled during this time. In the spatial probe test, a valid crossing of the PF was defined as the animal spending more than 0.1 s in the PF region. Occupancy of the target quadrant (TQ) was evaluated by the proportion of time or distance in the quadrant where the PF used to be located, calculated as: 


$$ \mathrm{Occupancy}=\frac{\mathrm{Time}\ \mathrm{or}\ \mathrm{travel}\ \mathrm{length}\ \mathrm{in}\ \mathrm{target}\ \mathrm{quadrant}}{\mathrm{Time}\ \left(\mathrm{60 \, s}\right)\ \mathrm{or}\ \mathrm{total}\ \mathrm{travel}\ \mathrm{length}\ \mathrm{in}\ \mathrm{probe}\ \mathrm{test}.} $$

### Surgery

All 12 tree shrews were subjected to a neurotoxic procedure to induce hippocampal lesions. The animals were anesthetized with isoflurane (airflow: 0.8–1.0 L/min, 0.5–3% isoflurane mixed with oxygen, adjusted according to physiological monitoring, RWD Life Science). Body temperature was maintained at ~38°C using a heating pad underneath the body with a closed-loop controller connected to a rectal temperature probe. Upon induction of anesthesia, Metacam (2 mg/mL, 1 mg/kg) and Baytril (50 mg/mL, 5 mg/kg) were injected subcutaneously, and the animal's head was fixed in a stereotaxic frame (RWD Life Science, China) with a custom-designed gas mask. Local anesthetic (2% Lidocaine, 200 μL) was applied under the skin before making the incision. Both temporalis muscles were gently detached from the skull and slightly moved such that infusion holes could be drilled in the skull above the hippocampus. A neurotoxin (colchicine, MedChemExpress, USA, CAS: 64-86-8, 0.6 mg/mL) dissolved in sterile phosphate-buffered saline (pH 7.2) was injected using a sharp 5 μL syringe (Hamilton, USA) mounted to the stereotaxic frame (needle opening directed caudally to the animal). The colchicine solution (1.0 μL) was infused at a speed of 10 μL/h using a micro pump (KD Scientific, USA), at 3 stereotaxic positions in the left and right hippocampi using bregma as a reference for infusion coordinates ((i) dorsal: AP +4.5 mm, ML ±5.0 mm, DV −4.0 mm, (ii) intermediate: AP +4.5 mm, ML ±7.0 mm, DV −7.0 mm, and (iii) ventral: AP +2.5 mm, ML ±4.5 mm, DV −11.2 mm). At each position, the needle was first lowered to the cranium below the injection site and then retracted 0.1 mm. The injection started 1 min after the needle was positioned. After the injection, the needle was left in place for 10 min to allow absorption. When the injections were completed, the skull was cleaned with saline solution, and the skin was sutured. The animals received postoperative care, including soft food, analgesics, and antibiotics for 3 days, followed by a 3-week recovery period with access to free food and water, before the second round of behavioral tests was performed.

### Histology

At the end of the experiment, the tree shrews were deeply anesthetized with an overdose of isoflurane, and transcardially perfused with 0.9% saline, followed by 10% formalin. After extraction from the skull, the brains were postfixed in 10% formalin overnight and subsequently in a 20, 30, and 30% sucrose sequence for 2–3 weeks until sectioning. Coronal sections (40 μm) were cut on a cryostat (KEDEE, KD-2950, China), with cresyl violet staining carried out on sections mounted on microscope slides (CITOGLAS, China). The sections were first dehydrated in graded ethanol baths (70, 80, 90, 100, 100, and 100%), cleared in turpentine oil, and rehydrated in a reverse direction in the same set of ethanol baths before staining with 0.1% cresyl violet solution (Sigma Aldrich, USA, CAS: 10510-54-0, Cat# C5042-10 g) for 5–10 min. The staining was differentiated by dipping the sections in a solution consisting of 70% ethanol and 0.5% acetic acid, after which the sections were dehydrated in ethanol baths and cleared in turpentine oil before being cover-slipped with neutral balsam. Finally, the Nissl-stained brain sections were imaged using a bright-field microscope (×10, 0.4 NA objective, Olympus, BX61, Japan), with OlyVIA software v3.2 (Build 21633, RRID: SCR_016167).

### Lesion quantification

To quantify the extent of hippocampal damage in each lesioned tree shrew, cell loss in each equally spaced brain section (120 μm) was identified. Regions of healthy hippocampal tissue (including the dentate gyrus, CA3, CA2, CA1, and subiculum) in each coronal section were manually labeled and measured using ImageJ software (RRID: SCR_003070; Schneider et al. 2012). The volume of healthy hippocampal formation was calculated by summing all labeled regions, then multiplying by the space between sections. The percentage of hippocampal damage was then determined by calculating the volume of lesioned tissue (derived by subtracting the volume of healthy hippocampal tissue from the volume of a standard hippocampus), expressed as a proportion of the volume of the standard hippocampus (78.10 mm^3^). This approach allowed quantitative assessment of the severity of hippocampal damage in each tree shrew and comparison of results across individuals.

### Statistical analysis

All results are presented as mean ± standard error of the mean (SEM). One-way ANOVA for repeated measures was applied to compare the learning curves before or after hippocampal lesions. Task performance before and after lesions was assessed by 2-way ANOVA for repeated measures, using a 2 × N design with both lesion and test day as within-subject factors. Bonferroni corrections were applied for multiple comparisons to reduce the likelihood of false positives. Wilcoxon signed-rank test and paired *t*-test were used to compare 2 groups of related measures. One sample *t*-test was used to compare the mean of measures with an expected value. Pearson correlation was used to evaluate the relationship between task performance and hippocampal damage. The level of significance was set to *P* < 0.05 and was given for 2-tailed tests. All statistical analyses were performed using SPSS statistics software v25 (IBM, RRID: SCR_002865).

## Results

### Radial-arm task

Tree shrews are skittish and agile animals, posing challenges for behavioral experiments. To overcome this, we first habituated the naïve animals to the laboratory environment and experimenters for 1 week, then trained and tested them in a novel radial-arm maze specifically tailored to the behavioral characteristics of tree shrews (Figs. 1 and S1a and b). In contrast to the rodent version, our radial-arm task necessitated that the tree shrews entered the center region from a designated start arm (arm 5) after the trial started and returned to the same arm after completing each trial. The maze design also allowed us to guide the animals to their destinations without provoking agitation. Cake rewards were placed in arms 2, 4, and 7, whereas the remaining 4 arms (1, 3, 6, and 8) were unbaited (Figs. S1a and 2A).

**Fig. 2:**
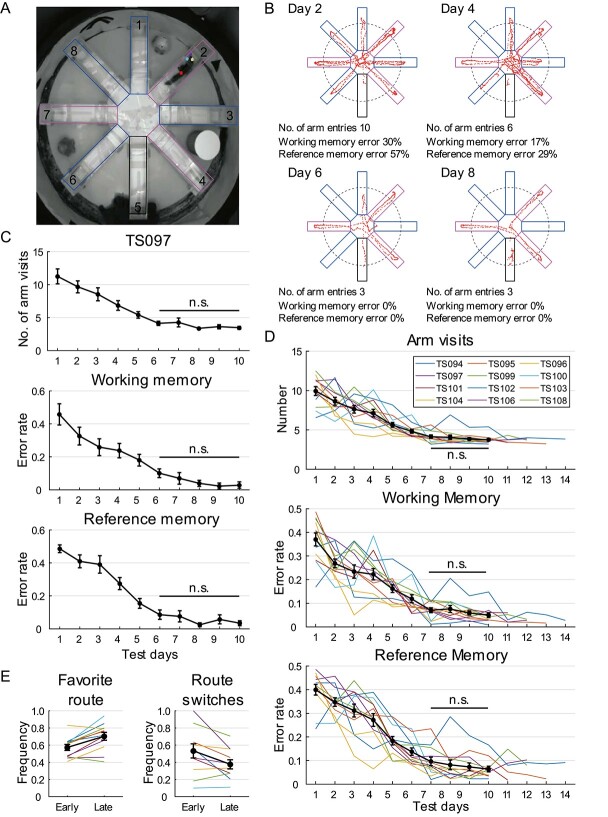
Tree shrew performance in radial-arm maze prior to hippocampal lesions. A) Camera footage of a tree shrew performing radial-arm task. Colored boxes denote maze arms for subsequent analysis. Black: start arm; magenta: baited arms; blue: unbaited arms. Arm IDs are indicated by numbers. Colored dots on animal are automatically labeled marks from DeepLabCut. B) Representative trials performed by TS097 in radial-arm task. Dots indicate location of snout. Dashed circle denotes threshold of a valid arm visit, counted when the snout position goes beyond the threshold. Colored boxes are the same as in panel a. C) Radial-arm maze task performance of TS097 during first 10 test days, assessed by number of arm visits (mean ± SEM) upon trial completion (top), error rates in WM (middle), and error rates in RM (bottom; 1-way ANOVA for repeated measures, arm entry: *F*(9) = 43.406, *P* < 0.001, *η*^2^ = 0.644; WM: *F*(9) = 28.115, *P* < 0.001, *η*^2^ = 0.539; RM: *F*(9) = 54.623, *P* < 0.001, *η*^2^ = 0.695). D) Tree shrew performance in radial-arm maze plateaued after day 7, as shown by number of arm visits (top), error rates in WM (middle), and error rates in RM (bottom). Black traces represent mean ± SEM, whereas colored lines indicate data from individual animals. E) Tree shrew preference for certain sequences after intensive training, measured by frequency of favorite route (left) and frequency of route switches (right). Color code is the same as in d. n.s., not significant.

During repeated training in the radial-arm maze, the tree shrews demonstrated a decrease in the number of visits to unbaited arms and a reduction in the number of revisits to baited arms over time (Fig. 2B). To measure the frequency of erroneous arm visits, we defined an arm visit as the nose position passing > 35 cm into the arm, which corresponds to the commonly used ×0.3 arm threshold for rodents (4 paws passing > 15 cm, plus a body length of 20 cm; Masuda et al. 1994). With continued training, the tree shrews showed a progressive decrease in the number of arms visited before consuming the 3 rewards, as well as a decline in both working memory (WM) error rates (frequency of repeated visits to arms) and reference memory (RM) error rates (frequency of visits to unbaited arms, which is spatial memory dependent; Fig. 2C). To determine the learning profiles of the tree shrews in the radial-arm paradigm, each animal was consecutively tested for at least 10 days, until both the WM and RM error rates were below 0.15, common criteria used in rodent behavioral experiments (Igarashi et al. 2014). After meeting these criteria, the animals were tested for 3 more days. Although there were noticeable individual differences in task acquisition, once the criteria were met, task performance remained stable over the following days, with error rates fluctuating slightly but never exceeding 0.15. Thus, we considered tree shrews to have learnt the radial-arm task once both error rates were <0.15. On average, the tree shrews learned the task after 6.83 ± 0.56 days (mean ± SEM).

We next quantified the performance of the 12 tree shrews in the radial-arm maze over 10 days using 3 measures: i.e. number of arm visits upon trial completion and error rates for WM and RM. A consistent and statistically significant decrease was observed in all 3 measures over the test days (1-way ANOVA for repeated measures, 12 animals, arm visits: *F*(9) = 39.904, *P* < 0.001, *η*^2^ = 0.784; WM: *F*(9) = 39.476, *P* < 0.001, *η*^2^ = 0.782; RM: *F*(9) = 51.746, *P* < 0.001, *η*^2^ = 0.825). Performance plateaued after 6 days of training, which was confirmed by post hoc analysis with Bonferroni correction (all *P* ≥ 0.795; Fig. 2D). The tree shrews also tended to develop a preference for a specific route when visiting the baited arms (stereotyped route pattern in consecutive trials), especially after they had mastered the task. This stereotypy was quantified by measuring the frequency of the favorite route in correct trials (trials without any erroneous arm visits) and frequency of route switches (proportion of correct trials in which a different route from the previous trial was selected). Results showed that the route preference increased significantly from 0.573 ± 0.035 in the early stage (test days 1–7) to 0.703 ± 0.045 in the late stage (test days 8–10; paired *t*-test, *t*(11) = 3.948, *P* = 0.002), whereas the frequency of switches decreased (from 0.533 ± 0.082 to 0.376 ± 0.053, *t*(11) = 2.747, *P* = 0.019; Fig. 2E), raising the possibility that the tree shrews developed alternative strategies to complete the task after extensive training. Based on our findings, we concluded that a 7-day test period was optimal for the tree shrew radial-arm task.

### Cheeseboard task

**Fig. 3:**
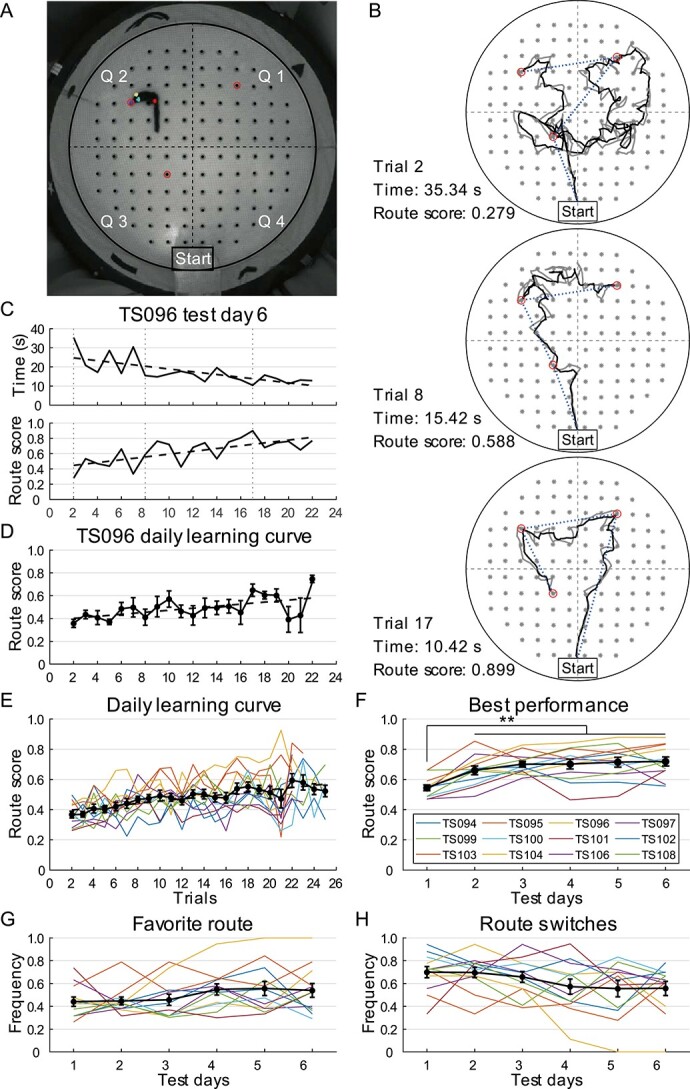
Tree shrew performance in cheeseboard maze prior to hippocampal lesions. A) Camera footage of a tree shrew performing cheeseboard task. Maze is partitioned into 4 quadrants using dashed lines. Baited wells are indicated by red circles. Colored dots on animal are automatically labeled marks from DeepLabCut. B) Representative trials performed by TS096 in cheeseboard maze on test day 6. Black and gray traces show trajectories of tree shrew head and snout, respectively. Positions of all 120 wells are marked with gray stars. Three baited wells are marked with circles. Blue dotted lines show distance between the start box and 3 baited wells, used to calculate route score. C) Learning curve of TS096 in cheeseboard maze on test day 6, evaluated by time spent (top) before consuming 3 rewards and route score (bottom). Dashed lines indicate learning trend. Vertical dotted lines refer to trials shown in panel b. D) Daily learning curve of TS096 (mean ± SEM) in cheeseboard maze, averaged across 6 test days (1-way ANOVA for repeated measures, *F*(20) = 2.566, *P* = 0.001, *η*^2^ = 0.339). E and F) Daily learning curve E) and best performance F) of individual tree shrews in cheeseboard maze, averaged across 6 test days and 5 best trials on each test day, respectively. Data from individual animals are shown using colored lines, whereas mean ± SEM of data from 12 animals is represented by black traces. G and H) Tree shrew route preference over 10 days, measured by frequency of favorite route G) and frequency of route switches H). Only TS104 displayed a clear preference over time. Same color code is used as in panel f. ^*^^*^, *P* < 0.01.

The tree shrews were next trained and tested using the cheeseboard maze (Fig. 1). Unlike the radial-arm task, where rewards were always baited in the same arms, a different set of reward wells was intentionally selected for the cheeseboard task each day (see Materials and Methods), thus requiring daily memory updates of goal locations. The distances between the 3 baited wells were kept comparable to maintain consistent levels of difficulty across days. With previous experience from the radial-arm maze, the tree shrews were more collaborative in the cheeseboard maze. They were easily lured back to the start box with a cake reward at trial end. The animals were tested in the cheeseboard maze for 6 consecutive days after learning to find rewards from 3 distant wells (Fig. 3A).

Over the 6-day test, the time spent and distance traveled to find the rewards, decreased in later trials (Fig. 3B). As the animals could visit the baited wells in different sequences and running speeds varied from trial to trial, “route score” was used to evaluate tree shrew memory for target locations in the maze. Route score was defined as the ratio between the ideal route to the 3 rewards and real distance traveled, with higher route scores indicating more accurate spatial memory. On each test day, the route score increased gradually with more trials, accompanied by a parallel decrease in trial time (Fig. 3C), indicating improvement in memory of reward locations with training. A similar increasing trend in route score was observed when the 6 test days were combined (Fig. 3D). Analysis of the daily learning curves of all 12 animals showed that cheeseboard maze performance improved progressively over 25 trials (1-way ANOVA for repeated measures, 12 animals, *F*(23) = 5.608, *P* < 0.001, *η*^2^ = 0.338; Fig. 3E).

Of note, in some animals, route scores decreased near the end of testing on each day, possibly because of diminished motivation to complete the task. Thus, the mean value of the best 5 trials (those with the highest route scores), rather than the last 5 trials, was calculated to represent the best performance an animal reached on each test day. Although some animals showed slight fluctuations in performance over the test days, group data indicated a rapid increase in performance that stabilized after the second day of training (1-way ANOVA for repeated measures, 12 animals, *F*(5) = 13.014, *P* < 0.001, *η*^2^ = 0.542, post hoc analysis with Bonferroni correction, test day 1 was significantly lower than other days, all *P* ≤ 0.003; Fig. 3F), whereas mean running speed remained unchanged over the test days (1-way ANOVA for repeated measures, *F*(5) = 0.737, *P* = 0.599, *η*^2^ = 0.063, *n* = 12; Supplementary Fig. S2b). We also investigated whether the animals developed any stereotypic behavior during the training sessions, which could potentially affect performance. We examined the frequency of the preferred route taken by each animal and the frequency of route switches but found no significant changes over the test days for most animals (1-way ANOVA for repeated measures, 12 animals, preferred route: *F*(5) = 1.733, *P* = 0.142, *η*^2^ = 0.136; route switches: *F*(5) = 2.101, *P* = 0.079, *η*^2^ = 0.160; Fig. 3G and H). These results suggest that the contribution of running speed and route preference to task performance was minimal and that cheeseboard maze performance was stable after 2 days of training.

### Water maze test

The tree shrews were trained and tested in the water maze after experiencing the dry-land mazes (Fig. 1). Extensive training in previous tests allowed the animals to be manually taken out from their nest boxes before each trial and carried from the PF and wiped dry with towels after each trial. Unlike previous tasks, where each tree shrew received custom pretraining until they met the criteria for everyday tests (see Materials and Methods), in the water maze test, all animals underwent the same pretraining, training and testing procedures, following rodent protocols (Vorhees and Williams 2006).

**Fig. 4:**
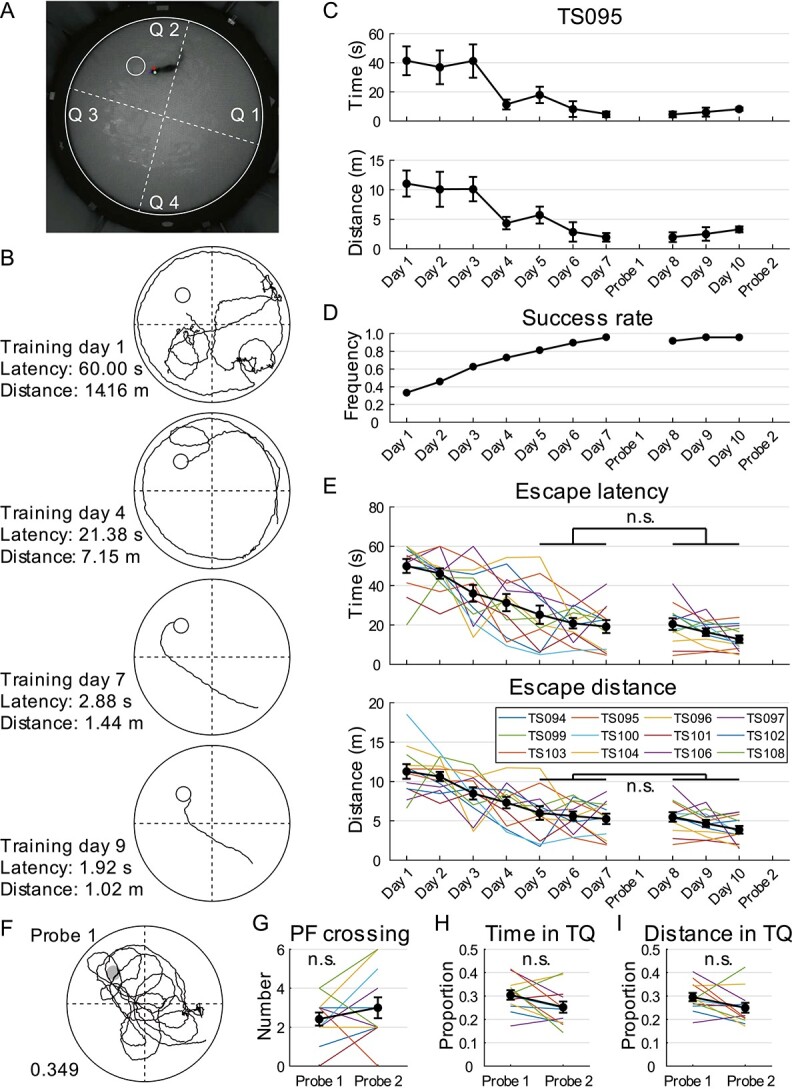
Tree shrew performance in water maze prior to hippocampal lesions. A) Camera footage of a tree shrew performing water maze test. PF location is indicated by smaller white circle and maze is divided into 4 quadrants by dashed lines. Colored dots on animal are automatically labeled marks from DeepLabCut. B) Representative training trials performed by TS095. The animal was released from the opposite quadrant. Trajectory of tree shrew head is indicated by black traces. PF is represented by a smaller black circle. C) Performance of TS095 in water maze during training was evaluated by time spent (mean ± SEM, top) and distance traveled (mean ± SEM, bottom) before reaching the PF (1-way ANOVA for repeated measures, time: *F*(6) = 3.685, *P* = 0.014, *η*^2^ = 0.551; distance: *F*(6) = 3.604, *P* = 0.016, *η*^2^ = 0.546). D) Success rate on each training day increased consistently before spatial probe tests. E) Tree shrew performance in water maze during training. Both escape latency (top) and escape distance (bottom) plateaued after 5 days. Data from individual animals are shown using colored lines, whereas mean ± SEM of data from 12 animals is represented by black traces. F) TS095 in the first spatial probe test. Trajectory of tree shrew head is indicated by black traces and PF location is represented by a dark circle. Number indicates distance proportion in TQ. This tree shrew crossed the PF location 3 times. G–I) Number of PF crosses G), percentage of time H), and travel distance I) in the TQ of each animal in the 2 spatial probe tests. Same color code is used as in panel e. n.s., not significant.

The 12 tree shrews were first pretrained with the PF visible on day 0, followed by 7 days of training with the PF hidden (days 1–7), after which their spatial memory was tested without the PF (probe 1). Unlike rats, tree shrews demonstrated vigorous swimming behavior to maintain their heads above water in the swimming pool, suggesting a poorer swimming ability compared with rats. Additionally, tree shrews faced challenges in sustaining swimming for longer than 1 min. Therefore, a 60-s trial limit was applied for the water maze test instead of the 2-min limits used for the dry-land tasks. During the first stage of training, tree shrews gradually learned to search for the hidden PF (Fig. 4A), successfully locating it after several days of training (Fig. 4B). This was evident from the decrease in time spent and distance traveled before reaching the PF (Fig. 4C). The success rate, defined as the proportion of trials where the animal successfully found the hidden PF, increased consistently from training days 1 to 7 (Fig. 4D), accompanied by a parallel increase in swimming speed (1-way ANOVA for repeated measures, *n* = 12, *F*(6) = 7.723, *P* < 0.001, *η*^2^ = 0.412; Supplementary Fig. S2c). During the first stage of training in the swimming pool, the spatial acquisition of the animals as a group exhibited a consistent decrease, as evidenced by the decline in escape latency and distance, defined as time spent and distanced traveled before locating the PF, respectively (1-way ANOVA for repeated measures, *n* = 12, latency: *F*(6) = 16.671, *P* < 0.001, *η*^2^ = 0.602; distance: *F*(6) = 11.512, *P* < 0.001, *η*^2^ = 0.511; Fig. 4E).

In the spatial probe test (probe 1), the trajectory of each animal was monitored for the first 60 s with the expectation that the tree shrews would explore the area and cross the PF location several times (Fig. 4F). To evaluate their probe test performance, 2 types of measures commonly used in rodents were used: i.e. number of PF location crossings (conservative measure) and occupancy time and distance in the TQ (sensitive measures; Maei et al. 2009). Group data analysis showed that the tree shrews crossed the PF 2.42 ± 0.34 times (Fig. 4G), and occupancy time and distance in the TQ were only slightly above a nonbiased search pattern (equal distribution of 0.25; time: 0.304 ± 0.020, 1-sample *t*-test, *n* = 12, *t* = 2.690, *P* = 0.021; distance: 0.295 ± 0.018, *t* = 2.528, *P* = 0.028; Fig. 4H and I). We attributed this to insufficient training before the probe test, and thus extended the swimming-pool task to 3 more days of training without changing the PF location (days 8–10), followed by a second spatial probe test (probe 2). The improvement in spatial learning during excess training was limited, with a trend toward statistical significance in both escape latency and distance when comparing days 8–10 with days 5–7 (2-way ANOVA for repeated measures, *n* = 12, latency: *F*(1, 2) = 4.579, *P* = 0.056, *η*^2^ = 0.294; distance: *F*(1, 2) = 3.677, *P* = 0.081, *η*^2^ = 0.251; Fig. 4D and E). Furthermore, PF location recall in the second probe test, evaluated by the number of PF crossings (3.00 ± 0.54), was not better than the first probe test (Wilcoxon signed rank test, *Z* = 1.021, *P* = 0.307; Fig. 4G), suggesting that additional training did not significantly improve memory recall of the PF location in the swimming pool. Notably, the TQ occupancy of tree shrews in probe test 2 decreased to an even distribution of 0.25 (time: 0.259 ± 0.025; distance: 0.257 ± 0.022; 1-sample *t*-test, both *P* ≥ 0.915), which, although not statistically different from probe test 1 (1-way ANOVA for repeated measures, *n* = 12, time: *F*(1) = 3.890, *P* = 0.074, *η*^2^ = 0.261; distance: *F*(1) = 3.263, *P* = 0.098, *η*^2^ = 0.229; Fig. 4H and I), suggests that TQ occupancy in probe tests may not be a dependable measure for evaluating the memory of tree shrews for the PF location. Overall, our study concluded that excessive training did not lead to significant improvements in spatial memory acquisition or retrieval in the swimming pool and that 7-day training is sufficient for tree shrews to learn the water maze test.

### Various hippocampal lesion sizes

To compare sensitivity to changes in spatial memory across different paradigms, we established hippocampal lesioning in the 12 tree shrews tested in all 3 mazes (Fig. 1). Colchicine infusions were administered to lesion the hippocampal formation, including the dentate gyrus, hippocampus proper, and subiculum (Keuker et al. 2003), along the dorsoventral axis in both hemispheres. We aimed to infuse the dorsal, intermediate, and ventral portions of the hippocampus on each side, with priority given to the dorsal hippocampus that is more involved in spatial memory (Moser et al. 1993, 1995; Moser and Moser 1998). The number of infusions depended on the state of the animal during surgery. Six tree shrews received the planned infusions at 6 infusion sites, whereas 5 tree shrews received 3–5 injections because of difficulties encountered during surgery (Table 1). One animal (TS104) died during the procedure, possibly because of isoflurane overdose, and was thus excluded from further analyses.

**Table 1:** Extent of hippocampal lesions in each tree shrew.

Animal	Left hippocampus			Right hippocampus			Total volume (mm^3^)	Mean lesion size (%)
	Injection sites	Volume (mm^3^)	Lesion size (%)	Injection sites	Volume (mm^3^)	Lesion size (%)		
TS094	3	15.56	80.08	3	12.70	83.74	28.25	81.91
TS095	3	6.66	91.47	3	3.01	96.15	9.67	93.81
TS096	2	24.18	69.04	2	29.78	61.87	53.96	65.46
TS097	1	54.30	30.47	2	31.03	60.27	85.33	45.37
TS099	3	6.98	91.06	3	10.97	85.95	17.95	88.51
TS100	1	59.11	24.31	2	32.32	58.61	91.43	41.46
TS101	3	6.08	92.22	3	5.74	92.65	11.82	92.43
TS102	3	4.07	94.79	3	3.61	95.38	7.68	95.08
TS103	2	34.06	56.39	2	36.48	53.28	70.54	54.84
TS106	3	8.62	88.97	3	25.49	67.36	34.11	78.16
TS108	3	6.67	91.46	2	25.77	67.00	32.44	79.23

**Fig. 5:**
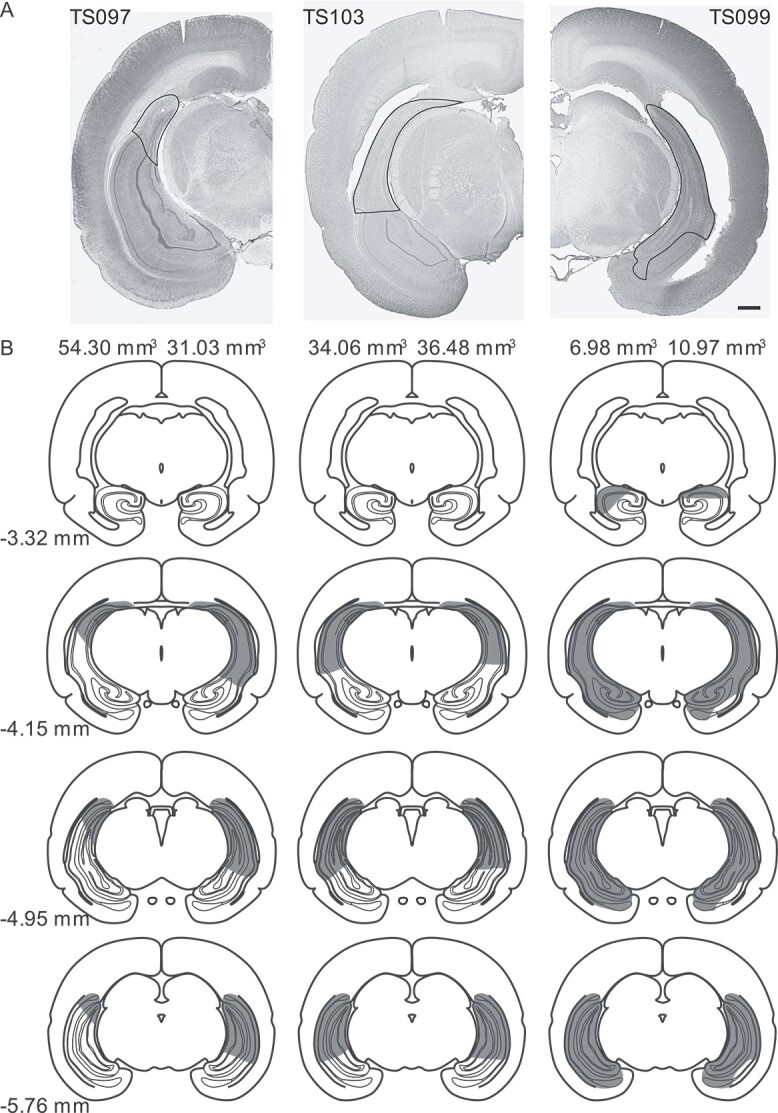
Representative lesions in the hippocampus. A) Brain sections from 3 animals with different levels of hippocampal lesions. Lesioned hippocampal tissue is shown using black traces. Scale bar: 1 mm. B) Representative schematics of equally spaced brain sections illustrating various degrees of hippocampal lesions in TS097 (left), TS103 (middle), and TS099 (right). Shadings indicate hippocampal lesions. Numbers on top of schematic indicate volume of healthy hippocampus in each hemisphere. Numbers at bottom left refer to distance posterior to Bregma. Brain profiles in panel b were adapted from *The tree shrew (Tupaia belangeri chinensis) brain in stereotaxic coordinates* (Zhou and Ni 2016) with permission.

After completing cognitive tests, histological samples were obtained to assess the extent of the hippocampal lesions in the remaining animals. The border between the damaged and healthy hippocampal tissue was sharp and clearly defined (Fig. 5A). The volume of healthy hippocampal tissue was quantified for each animal and the proportion of lesioned tissue was estimated based on a standard tree shrew hippocampus (volume 78.10 mm^3^; Fig. 5B). The volume of healthy tissue varied among animals, ranging from 7.68 to 91.43 mm^3^, corresponding to lesion sizes between 41.46 and 95.08% (Table 1). The remaining sizes of individual subregions were largely proportional to total volume (Supplementary Tables S1 and S2). Minor inadvertent damage was observed in para-hippocampal regions, including the entorhinal cortex and pre- and para-subiculum, in TS095 and TS102, which exhibited the most effective hippocampi lesions. An unintended cortical lesion was also observed in TS106, possibly resulting from unsuccessful injections to the right hippocampus, which damaged the piriform cortex but spared part of the dorsal dentate gyrus (Supplementary Fig. S3).

### Degenerated RM in radial-arm task after hippocampal lesion

In total, 11 tree shrews were tested in the radial-arm maze at least 3 weeks after lesions to both hippocampi (Fig. 1). Arms 3, 6, and 8 were baited after the lesions, which had a symmetrical spatial arrangement to the baited arms in the previous test. The animals were tested for 7 consecutive days, with similar numbers of trials performed as in the previous tests. Of the 11 animals, 10 exhibited proficient performance in the task, whereas TS106 displayed persistent high error rates in both WM and RM over the test days following the establishment of lesions (1-way ANOVA for repeated measures for TS106, 25 trials, WM: *F*(6) = 1.158, *P* = 0.332, *η*^2^ = 0.046; RM: *F*(6) = 1.623, *P* = 0.145, *η*^2^ = 0.063; other animals: all *P* ≤ 0.002). This poor performance may be attributed to sudden weight gain during the post-lesion recovery period, which compromised task performance motivation (Supplementary Fig. S2a). Thus, TS106 was removed from further analysis in the radial-arm maze. The frequencies of the preferred route and route switches when visiting baited arms remained relatively stable and did not exhibit significant changes after the lesion (test days 1–7, preferred route: pre-lesion: 0.599 ± 0.036, post-lesion: 0.561 ± 0.066, paired *t*-test, *t*(9) = 0.493, *P* = 0.634; route switches: pre-lesion: 0.473 ± 0.082, post-lesion: 0.481 ± 0.159, paired *t*-test, *t*(9) = 0.113, *P* = 0.912), indicating that the tree shrews completed both rounds of radial-arm tasks using the same strategy, in the first 7 days.

**Fig. 6:**
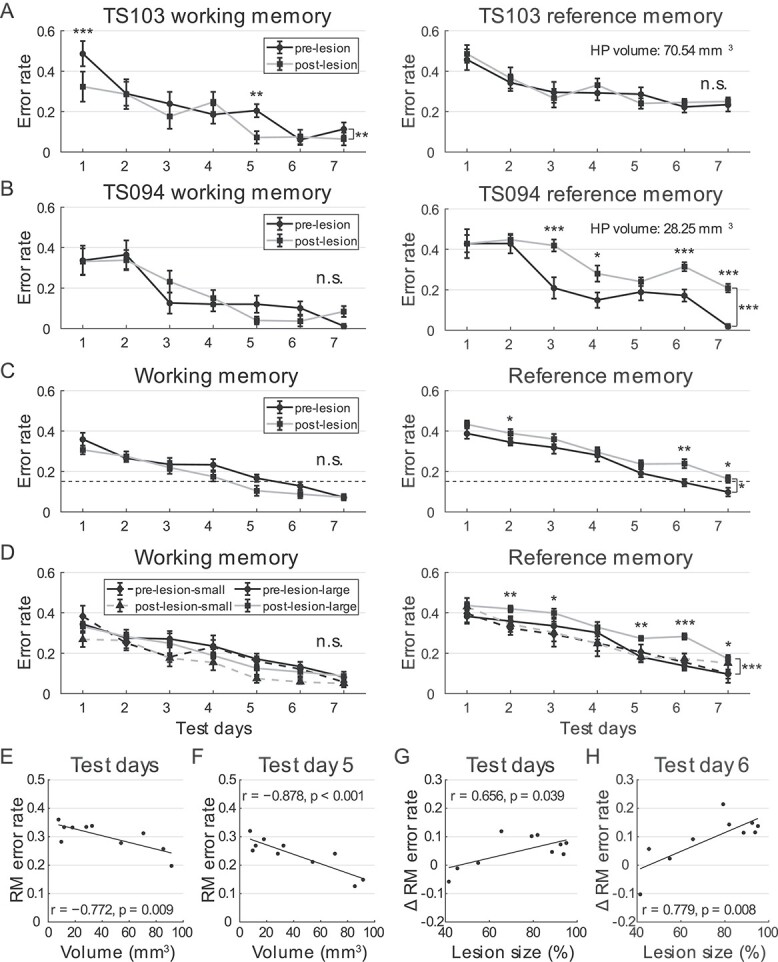
Tree shrew performance in radial-arm maze after hippocampal lesions. A and B) WM (mean ± SEM, left) and RM (mean ± SEM, right) performance in tree shrews with small A) or large hippocampal lesions B). TS103 exhibited a slight increase in WM performance on test days 1 and 5 (2-way ANOVA for repeated measures, test day: *F*(6) = 18.095, *P* < 0.001, *η*^2^ = 0.430; lesion: *F*(1) = 8.218, *P* = 0.008, *η*^2^ = 0.255; lesion × test day: *F*(1, 6) = 3.678, *P* = 0.002, *η*^2^ = 0.133), whereas RM remained unchanged (test day: *F*(6) = 17.539, *P* < 0.001, *η*^2^ = 0.422; lesion: *F*(1) = 0.227, *P* = 0.638, *η*^2^ = 0.009; lesion × test day: *F*(1, 6) = 0.874, *P* = 0.516, *η*^2^ = 0.035). WM in TS094 was not altered (test day: *F*(6) = 27.555, *P* < 0.001, *η*^2^ = 0.534; lesion: *F*(1) = 0.136, *P* = 0.716, *η*^2^ = 0.006; lesion × test day: *F*(1, 6) = 3.040, *P* = 0.008, *η*^2^ = 0.112), but RM was impaired after lesion (test day: *F*(6) = 45.539, *P* < 0.001, *η*^2^ = 0.655; lesion: *F*(1) = 51.936, *P* < 0.001, *η*^2^ = 0.684; lesion × test day: *F*(1, 6) = 5.410, *P* < 0.001, *η*^2^ = 0.184). C) WM (left) and RM (right) of tree shrews included in the analysis before and after lesion (mean ± SEM). Dashed lines indicate 0.15 threshold for task learning. Note, RM error rates were consistently higher than 0.15 post-lesion. D) Task performance in radial-arm maze of animals grouped by volume of healthy hippocampal tissue (mean ± SEM). E) Scatter plots showing significant correlations between volume of healthy hippocampal tissue and RM error rates averaged across 7 test days post-lesion. Regression line is in black. F) Same as in panel e, but showing data for post-lesion test day 5, when the correlation was most prominent. G) Scatter plots showing significant correlation between extent of hippocampal lesions and changes in RM over the course of 7 test days post-lesion. H) Same as in panel g, but showing data on post-lesion test day 6, when the difference in RM was most pronounced. n.s., not significant, ^*^, *P* < 0.05, ^*^^*^, *P* < 0.01, ^*^^*^^*^, *P* < 0.001.

We first compared WM on pre- and post-lesion test days 1–7, and found no impairment after the lesion (Fig. 6A and B, left). Analysis of group data indicated that WM performance significantly improved over the test days after hippocampal lesion, similar to the pre-lesion trials (2-way ANOVA for repeated measures, 10 animals, *F*(6) = 30.775, *P* < 0.001, *η*^2^ = 0.774). No significant differences in WM performance were observed before and after the lesion (*F*(1) = 2.895, *P* = 0.123, *η*^2^ = 0.243) and no interactions were found between hippocampal lesion and test days (*F*(1, 6) = 1.782, *P* = 0.120, *η*^2^ = 0.165; Fig. 6C, left). While the role of the hippocampus in WM continues to be a subject of debate, our findings are consistent with recent systematic analysis of 26 human studies, suggesting that the hippocampus does not play a significant role in WM (Slotnick 2022). In contrast, however, our findings indicated that RM remained unchanged in tree shrews with relatively small hippocampal damage but was impaired when both hippocampi were extensively compromised (Fig. 6A and B, right). Further analysis of group data indicated that RM after the lesion improved with test days but was significantly impaired (2-way ANOVA for repeated measures, 10 animals, test day: *F*(6) = 45.159, *P* < 0.001, *η*^2^ = 0.834; lesion: *F*(1) = 7.717, *P* = 0.021, *η*^2^ = 0.462), with maximum change on day 6 (post hoc analysis with Bonferroni correction). Again, no lesion and test day interactions were observed (*F*(1, 6) = 1.090, *P* = 0.380, *η*^2^ = 0.108; Fig. 6C, right). These findings suggest that the hippocampus plays a critical role in RM, but not in WM, when tree shrews performed the radial-arm task.

To further clarify the relationship between hippocampal function and RM, we divided the tree shrews into 2 groups based on the size of their healthy hippocampal tissue. The first group had small hippocampal lesions (volume > 50 mm^3^), whereas the second group had large lesions (volume < 50 mm^3^). Results showed that both groups exhibited unchanged WM post-lesion, as demonstrated by 2-way ANOVA for repeated measures (small-lesion group: 4 animals, test day: *F*(6) = 12.618, *P* < 0.001, *η*^2^ = 0.808; lesion: *F*(1) = 2.209, *P* = 0.234, *η*^2^ = 0.424; lesion × test day: *F*(1, 6) = 2.221, *P* = 0.089, *η*^2^ = 0.425; large-lesion group, 6 animals, test day: *F*(6) = 16.648, *P* < 0.001, *η*^2^ = 0.769; lesion: *F*(1) = 0.752, *P* = 0.425, *η*^2^ = 0.131; lesion × test day: *F*(1, 6) = 0.528, *P* = 0.782, *η*^2^ = 0.096; Fig. 6D, left). Visits to unbaited arms were not affected in the small-lesion group after hippocampal lesion (2-way ANOVA for repeated measures, 4 animals, test day: *F*(6) = 12.890, *P* < 0.001, *η*^2^ = 0.811; lesion: *F*(1) = 0.153, *P* = 0.722, *η*^2^ = 0.049; lesion × test day: *F*(1, 6) = 0.291, *P* = 0.933, *η*^2^ = 0.089), but increased significantly in the large-lesion group (2-way ANOVA for repeated measures, 6 animals, test day: *F*(6) = 34.871, *P* < 0.001, *η*^2^ = 0.875; lesion: *F*(1) = 42.217, *P* = 0.001, *η*^2^ = 0.894; lesion × test day: *F*(1, 6) = 2.119, *P* = 0.080, *η*^2^ = 0.298) on 5 of the 7 test days (post hoc analysis with Bonferroni correction). The same trends held true in normalized measures (Supplementary Fig. S4b).

We found a strong relationship between size of healthy hippocampal tissue and RM in the tree shrews, as evidenced by significant correlations between remaining tissue volume and RM error rates averaged across post-lesion test days (Fig. 6E). Similar correlations were also observed between areas of individual subregions and RM (Supplementary Table S2). Further analysis revealed that this volume–performance correlation was also significant on test days 3–6 (Pearson correlation, *P* ≤ 0.03; Fig. 6F). We also investigated whether the extent of hippocampal damage affected spatial memory and found that the reduction in RM was indeed associated with lesion size (Fig. 6G). This correlation was strongest on test day 6, when the largest difference in spatial memory was observed (Fig. 6H). Taken together, these findings indicate that tree shrew RM in the radial-arm maze was impaired by significant hippocampal lesions, whereas WM remained largely unaffected. The declines in RM were also correlated with hippocampal lesion size. These conclusions were supported by analyses that included TS106 (data not shown).

### Dramatic declines in post-lesion spatial learning in cheeseboard maze

**Fig. 7:**
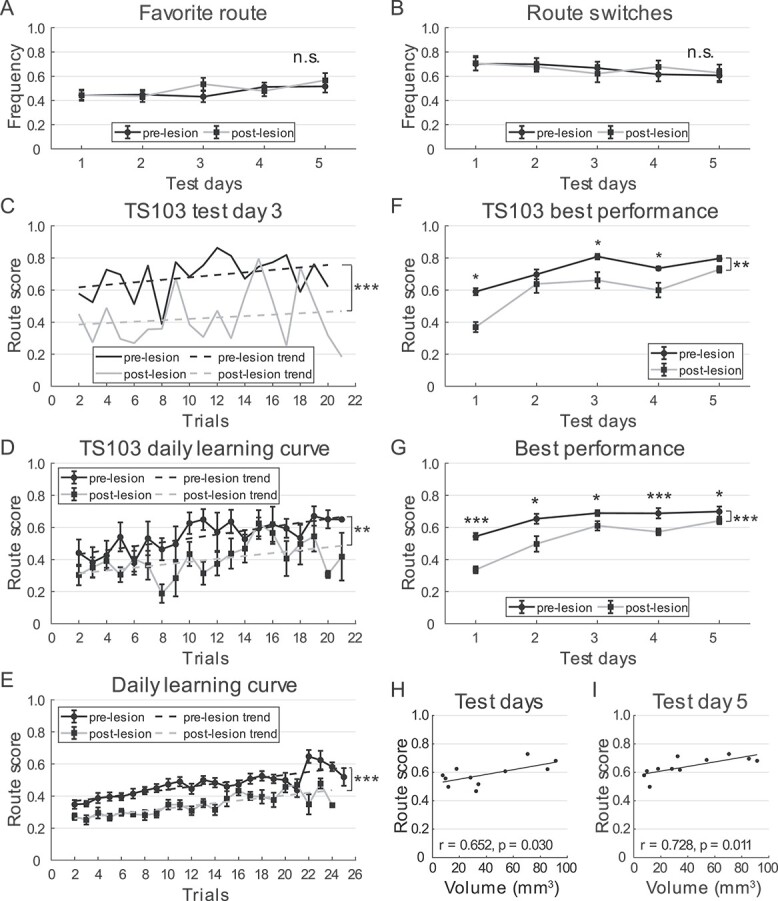
Tree shrew performance in cheeseboard maze after hippocampal lesions. A and B) Tree shrew route preference on pre- and post-lesion days, measured by frequency of favorite route (mean ± SEM, A) and frequency of route switches (mean ± SEM, B). C) Learning curve of TS103 on test day 3, before and after lesions (paired *t*-test, *t*(19) = 6.053, *P* < 0.001). D) Daily learning curve of TS103 (mean ± SEM), averaged across 5 test days (2-way ANOVA for repeated measures, trial: *F*(19) = 2.781, *P* = 0.001, *η*^2^ = 0.410; lesion: *F*(1) = 22.307, *P* = 0.009, *η*^2^ = 0.848; lesion × trial: *F*(1, 19) = 1.337, *P* = 0.186, *η*^2^ = 0.251). E) Daily learning curve (mean ± SEM) of tree shrews in cheeseboard maze, averaged across 5 test days. F) Best performance of TS103 (mean ± SEM) before and after lesions, averaged from 5 best trials on each test day (2-way ANOVA for repeated measures, test day: *F*(4) = 19.192, *P* < 0.001, *η*^2^ = 0.828; lesion: *F*(1) = 38.068, *P* = 0.004, *η*^2^ = 0.905; lesion × test day: *F*(1, 4) = 2.188, *P* = 0.117, *η*^2^ = 0.354). G) Same as in panel f, but best performance (mean ± SEM) on each test day averaged across all animals. H) Scatter plots showing significant correlation between volume of healthy hippocampal tissue and best performance averaged across 5 test days after lesions. Regression line is in black. I) Same as in panel h, but showing correlation between residual hippocampal volume and best performance on post-lesion test day 5. n.s., not significant, ^*^, *P* < 0.05, ^*^^*^, *P* < 0.01, ^*^^*^^*^, *P* < 0.001.

The hippocampal-lesioned tree shrews were next retested in the cheeseboard maze (Fig. 1). To ensure consistency between the 2 rounds of tests, the wells were baited in a manner similar to that in the pre-lesion tests (see Materials and Methods). Although group data from the previous test plateaued from day 3 (Fig. 3F), individual animal performance fluctuated over days. Therefore, the animals were tested for 5 consecutive days. Similar to previous research in rats (Olton and Werz 1978), the tree shrew running speed in the maze was higher after hippocampal lesion, especially on early test days (Supplementary Fig. S2b), suggesting that locomotion ability was not impaired by the lesions. Moreover, the animals employed the same strategy to complete the tasks, as evidenced by the consistent degree of route preference in both test rounds (2-way ANOVA for repeated measures, 11 animals, frequency of favorite sequence: test day: *F*(4) = 1.884, *P* = 0.132, *η*^2^ = 0.159; lesion: *F*(1) = 0.426, *P* = 0.529, *η*^2^ = 0.041; lesion × test day: *F*(1, 4) = 1.021, *P* = 0.408, *η*^2^ = 0.093; frequency of sequence switches: test day: *F*(4) = 1.005, *P* = 0.417, *η*^2^ = 0.091; lesion: *F*(1) = 0.010, *P* = 0.922, *η*^2^ = 0.001; lesion × test day: *F*(1, 4) = 0.391, *P* = 0.814, *η*^2^ = 0.038; Fig. 7A and B).

The animals were able to partially remember the locations of reward wells after undergoing more than 20 training trials, but their routes to the rewards were always longer than those on the same test day prior to the lesion (Fig. 7C). Combining test days also revealed a significant reduction in route scores (Fig. 7D). Analysis of the learning curves of all animals revealed that their memory of reward locations in the cheeseboard maze gradually improved within 25 trials in both rounds of tests (2-way ANOVA for repeated measures, 11 animals, *F*(22) = 13.291, *P* < 0.001, *η*^2^ = 0.571), but was significantly impaired after bilateral hippocampal lesion (*F*(1) = 64.935, *P* < 0.001, *η*^2^ = 0.867). A significant trial and lesion interaction was also observed (*F*(1, 22) = 3.684, *P* < 0.001, *η*^2^ = 0.269; Fig. 7E). Best performance was significantly reduced after lesion for each tree shrew (2-way ANOVA for repeated measures, all *P* ≤ 0.036; Fig. 7F). Analysis of group data also revealed a significant reduction in best performance (2-way ANOVA for repeated measures, 11 animals, test day: *F*(4) = 59.011, *P* < 0.001, *η*^2^ = 0.855; lesion: *F*(1) = 44.780, *P* < 0.001, *η*^2^ = 0.817; lesion × test day: *F*(1, 4) = 3.759, *P* = 0.011, *η*^2^ = 0.273), significant on every test day (post hoc analysis with Bonferroni correction; Fig. 7G). Similar results were obtained using normalized measures (Supplementary Fig. S4b).

We next asked whether post-lesion task performance was affected by hippocampal lesion size. Analysis revealed a significant correlation between the size of healthy hippocampal tissue and average routes cores (Fig. 7H and Supplementary Table S2), which was most pronounced on test day 5 (Fig. 7I). These results indicate that spatial learning in tree shrews in the cheeseboard task was markedly impaired by bilateral hippocampal lesions, even in animals with the smallest damage, raising the possibility that this paradigm is sensitive to subtle changes in spatial memory.

### Impaired spatial memory retrieval in water maze test after lesion

The tree shrews were subjected to a final water maze test (Fig. 1). The PF was moved from quadrants 2 to 3 for this testing. Surprisingly, unlike the other 2 tasks, the tree shrews performed much better after the lesion, with similar swimming pool performance on post-lesion days 1–4 and pre-lesion days 4–7. Under such circumstances, it was deemed unnecessary to compare spatial learning (memory encoding) in the water maze before and after the lesion. Therefore, we modified the protocol by testing the tree shrews after 4 days of training (probe 1) when they displayed comparable escape behavior. Following the first probe test, the animals were trained for an additional 3 days without relocating the PF (days 5–7), with memory retrieval tested for a second time (probe 2), similar to the previous experiment.

**Fig. 8:**
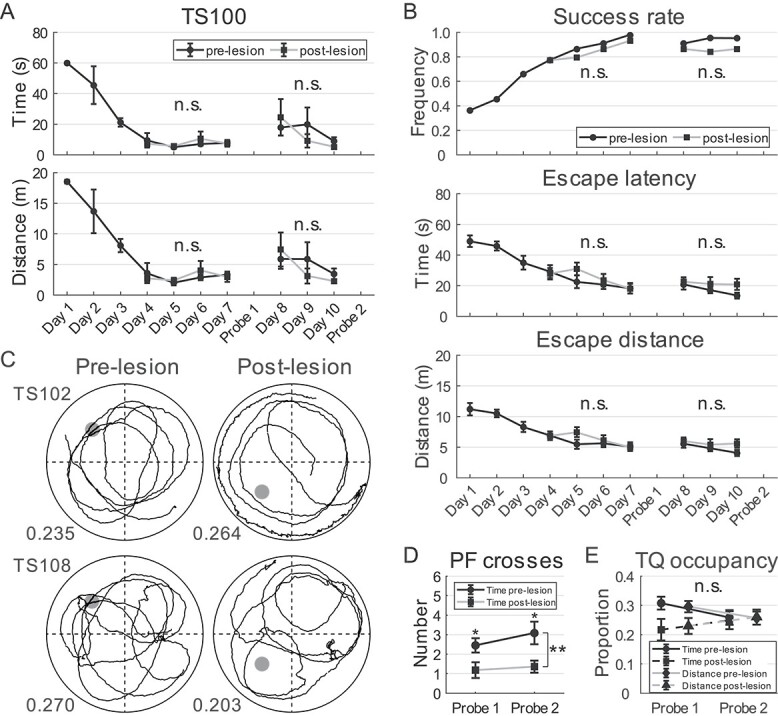
Tree shrew performance in water maze after hippocampal lesions. A) Performance of TS100 on training days before and after lesions, measured by escape latency (mean ± SEM, top) and distance (mean ± SEM, bottom). Post-lesion days 4–10 in the plots were training days 1–7 after lesion. Both time and distance were comparable after realignment (2-way ANOVA for repeated measures, all *P* ≥ 0.232). B) Success rate (top), escape latency (mean ± SEM, middle), and distance (mean ± SEM, bottom) on pre- and post-lesion training days. C) Trajectories of TS102 and TS108 in probe test 1, before and after hippocampal lesions, indicated by black traces. PF location is represented by a dark circle. Numbers indicate distance proportion in TQ. D and E) Number of PF crosses (mean ± SEM, D) and TQ occupancy (mean ± SEM, E) in spatial probe tests before and after lesions. n.s., not significant, ^*^, *P* < 0.05, ^*^^*^, *P* < 0.01.

For the sake of convenience, post-lesion training days 1–7 were aligned with pre-lesion training days 4–10 in the analyses. The individual learning curves of the animals post-lesion did not differ significantly from those during pre-lesion training in the 2 training stages (2-way ANOVA for repeated measures, all *P* ≥ 0.139; Fig. 8A). Group data indicated that the training trial success rates were close to statistical significance after lesion (paired *t*-test, first stage: *t*(3) = 2.784, *P* = 0.069; second stage: *t*(2) = 4.150, *P* = 0.053). Both escape latency and distance decreased over time during the first stage of training (2-way ANOVA for repeated measures, 11 animals, latency: *F*(3) = 6.164, *P* = 0.002, *η*^2^ = 0.381; distance: *F*(3) = 3.316, *P* = 0.040, *η*^2^ = 0.239), but not in the second stage (latency: *F*(2) = 1.943, *P* = 0.169, *η*^2^ = 0.163; distance: *F*(2) = 1.922, *P* = 0.172, *η*^2^ = 0.161). No significant differences were observed between the pre- and post-lesion training days in escape latency (first stage: *F*(1) = 1.100, *P* = 0.319, *η*^2^ = 0.099; second stage: *F*(1) = 2.601, *P* = 0.138, *η*^2^ = 0.206) or escape distance (first stage: *F*(1) = 1.122, *P* = 0.314, *η*^2^ = 0.101; second stage: *F*(1) = 1.820, *P* = 0.207, *η*^2^ = 0.154). No lesion and training day interactions were detected in the tests (first stage: latency: *F*(1, 3) = 1.668, *P* = 0.195, *η*^2^ = 0.143; distance: *F*(1, 3) = 1.493, *P* = 0.237, *η*^2^ = 0.130; second stage: latency: *F*(1, 2) = 0.964, *P* = 0.398, *η*^2^ = 0.088; distance: *F*(1, 2) = 1.075, *P* = 0.360, *η*^2^ = 0.097; Fig. 8B). These results indicate that spatial memory acquisition was comparable before the retrieval tests, suggesting that even a small portion of remaining hippocampal tissue can support spatial learning in the water maze. These findings in tree shrews are consistent with previous studies in rats (Moser et al. 1993, 1995; Moser and Moser 1998).

We next compared retrieval of spatial memory in tree shrews on the probe tests. The number of PF crossings declined in some animals after the lesion, whereas TQ occupancy remained largely unchanged (Fig. 8C). Analysis of group data showed that the number of PF crossings was similar between the first and second probe tests (post-lesion test 1: 1.18 ± 0.40; test 2: 1.36 ± 0.31; 2-way ANOVA for repeated measures, 11 animals, *F*(1) = 1.000, *P* = 0.341, *η*^2^ = 0.091), but was significantly reduced post-lesion (*F*(1) = 12.692, *P* = 0.005, *η*^2^ = 0.559) in both tests (post hoc analysis with Bonferroni correction; Fig. 8D). No lesion and test interaction was detected (*F*(1, 1) = 0.387, *P* = 0.548, *η*^2^ = 0.037). TQ occupancy by the tree shrews in both probe tests post-lesion was very close to an equal distribution of 0.25 (1-sample *t*-test, both *P* ≥ 0.392). No differences were observed in occupancy post-lesion (time: lesion: *F*(1) = 3.246, *P* = 0.102, *η*^2^ = 0.245; test: *F*(1) = 0.142, *P* = 0.714, *η*^2^ = 0.014; lesion × test: *F*(1, 1) = 1.393, *P* = 0.265, *η*^2^ = 0.122; distance: lesion: *F*(1) = 2.233, *P* = 0.166, *η*^2^ = 0.183; test: *F*(1) = 0.046, *P* = 0.834, *η*^2^ = 0.005; lesion × test: *F*(1, 1) = 1.510, *P* = 0.247, *η*^2^ = 0.131; Fig. 8E), suggesting that tree shrews may employ a different strategy than rodents when searching for the PF. Different from the other 2 tasks, no correlation was observed between hippocampal lesion size and spatial learning during training or recall of PF location in the probe tests (Pearson correlation, 11 animals, all *P* ≥ 0.115). These results suggest that recall of the PF location in the swimming pool was impaired after hippocampal lesion, even when comparable levels of spatial learning had been achieved. This implies a potential dissociation between encoding and retrieval of spatial memory in the hippocampus, consistent with previous reports in both rats and humans (Zeineh et al. 2003; Lee and Kesner 2004; Eldridge et al. 2005; Duncan et al. 2014).

## Discussion

Tree shrews are considered a viable animal model for research on AD (Fan et al. 2018), a neurodegenerative disease characterized by impaired spatial cognition during its early stages (Coughlan et al. 2018). Currently, cognitive paradigms to evaluate spatial memory in tree shrews are limited. In this study, we developed several paradigms to assess spatial memory in tree shrews and compared their robustness by testing 12 animals before and after the creation of bilateral hippocampal lesions. Results indicated that hippocampal lesions compromised task performance in all mazes, but the degree of deterioration varied across paradigms. Our findings showed that the cheeseboard task was the most appropriate choice among the 3 spatial paradigms for evaluating spatial memory impairment in tree shrews and may potentially help to monitor progressive cognitive declines in aging and disease models.

### Landmark-based navigation in spatial tasks

Behavioral paradigm testing spatial memory typically requires animals to navigate mazes based on distal landmarks (visuospatial navigation), rather than local cues (beacon navigation; Nyberg et al. 2022). To achieve this, we tested the tree shrews in a well-lit room enriched with distal visual cues, removed all possible visual and tactile marks near the rewards, and obscured reward odor to minimize local cues (see Materials and Methods). Initially, the tree shrews struggled not to follow the odor cues but soon learned to ignore them during pretraining, indicating that they learnt to use distal visual cues rather than proximal odor cues to locate rewards. This was further supported by observations from another group of 4 animals in our laboratory. We conducted tests using a cue-deprived version of the radial-arm maze, where distal landmarks were largely not visible. The tree shrews were unable to locate the baited arms even after 3 weeks of training, instead developing a kinesthetic strategy to visit adjacent arms consecutively (data not shown). Therefore, our results indicate that the tree shrews used distal landmarks to position themselves within the mazes, suggesting the involvement of their internal navigation system when performing the tasks.

### Varied spatial memory demand across behavioral paradigms

In the present study, we compared the spatial memory of tree shrews before and after hippocampal lesion to avoid potential inter-individual behavioral differences. This experimental design allowed each animal to serve as its own control and enabled comparison the 3 paradigms within the same animal. Results showed that spatial learning in the cheeseboard and radial-arm mazes was compromised post-lesion (Figs. 6C, 7E and G), whereas memory retrieval was impaired in the water maze test (Fig. 8C and D). Moreover, compromised spatial learning was observed in the cheeseboard task but not in the radial-arm task in tree shrews with relatively small hippocampal damage (Figs. 6A, 7C, D and F). Overall, our findings suggested that the cheeseboard maze was the most sensitive to loss of spatial memory among the 3 paradigms. Importantly, the minimal influence of previous experiences on the cheeseboard maze and its reservoir of reward locations facilitated multiple repetitions of the task in the same animals, which is crucial for monitoring progressive changes in spatial memory during aging and the development of AD.

The differential effects of hippocampal damage on these paradigms may be attributed to varying levels of spatial coding in the entorhinal-hippocampal circuit. Specifically, we observed that animals were required to memorize 3 target locations in both the cheeseboard and radial-arm mazes, but only 1 PF location in the water maze. This difference suggests that more hippocampal neurons may be recruited to represent multiple goal locations in reward-based paradigms (Dupret et al. 2010; Sarel et al. 2017; Spiers et al. 2018). The cheeseboard paradigm also required animals to daily update reward locations, which may be stabilized by short inter-trial hippocampal replays, whereas in the other 2 tasks where target locations remained static, spatial memory initially encoded in the hippocampus may be enhanced and transmitted to the neocortex during sleep by post-training consolidation (Klinzing et al. 2019; Brodt et al. 2023). Therefore, hippocampal lesions may be partially compensated by memory consolidation in the radial-arm and swimming-pool tasks, but not in the cheeseboard task. Finally, animals in the cheeseboard maze had to compute both distance and direction to locate reward positions, whereas in the radial-arm maze only direction was necessary to identify the baited arms. Studies have shown that the processing of distance and direction coding occurs independently through separate populations of neurons located in distinct layers of the medial entorhinal cortex (Fyhn et al. 2004; Hafting et al. 2005; Sargolini et al. 2006), which project both locally and to different subregions of the hippocampus (Canto et al. 2008). Based on our observations, we suggest that the convergence of distance and direction information is more likely to occur in the hippocampus than in the entorhinal cortex, as the integrity of the hippocampus was critical for completing the cheeseboard task.

### Interspecies variability in the water maze

**Fig. 9:**
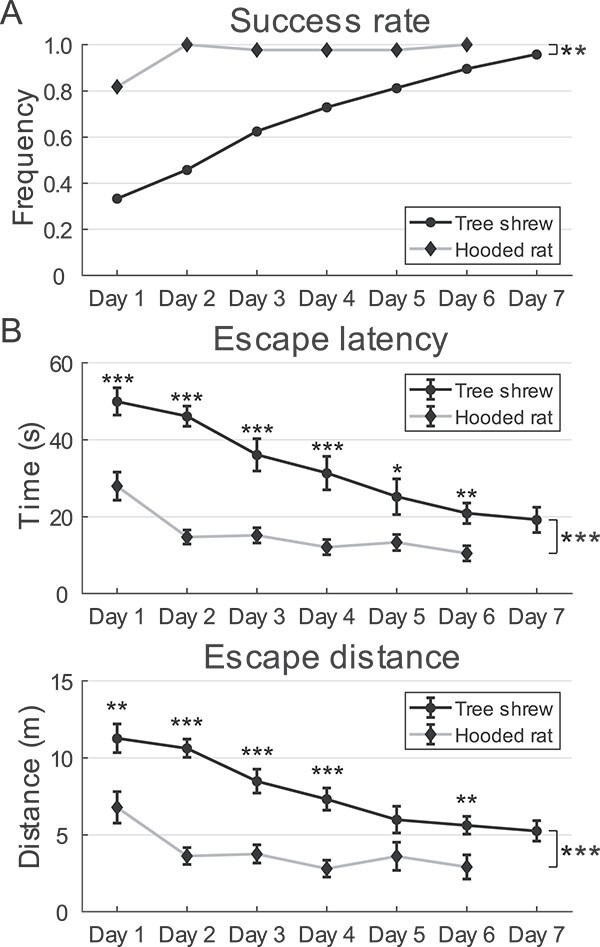
Fast learning of hooded rats in water maze. A) Success rates of rats and tree shrews on each training day. Rats showed significantly higher success rates than tree shrews (paired *t*-test, *t*(5) = 4.423, *P* = 0.007). B) Water maze learning curves (mean ± SEM) during training for both species. Rats found the PF significantly faster than tree shrews, reflected by both escape latency (top) and escape distance (bottom; 2-way ANOVA for repeated measures, 23 animals, escape latency: species: *F*(1) = 41.544, *P* < 0.001, *η*^2^ = 0.664; training day: *F*(5) = 20.489, *P* < 0.001, *η*^2^ = 0.494; training day × species: *F*(1, 5) = 4.207, *P* = 0.002, *η*^2^ = 0.167; escape distance: species: *F*(1) = 40.543, *P* < 0.001, *η*^2^ = 0.659; training day: *F*(5) = 15.136, *P* < 0.001, *η*^2^ = 0.419; training day × species: *F*(1, 5) = 3.301, *P* = 0.008, *η*^2^ = 0.136).

In this study, tree shrews exhibited faster learning of the PF location in the second round of water maze testing, even after bilateral hippocampal lesions (Fig. 8A and B). This suggests that previous experience strongly influences spatial learning in repeated water maze tests. Furthermore, our observations indicated that tree shrews may be less adept at swimming than rats, which may partially explain why more practice is required before they can effectively navigate using distal landmarks in water-based tasks (Whishaw and Tomie 1996). To further support this hypothesis, we compared the performances of 11 male Long-Evans rats of similar age (22 weeks) using the same swimming pool and training protocol (Fig. 9). The results showed that rats learned the PF location significantly faster than tree shrews. However, the dry-land task results indicated that the impairments observed in the same group of tree shrews in the swimming pool were not because of inadequacy in spatial memory *per se*, but rather to other nonspatial limiting factors such as swimming ability. Variations in swimming skills may also lead to different search strategies in the water maze. Notably, we observed that tree shrew occupancy in the TQ during the probe tests exhibited a near unbiased search pattern (Figs. 4H, I, 8C and E), differing from previous reports in rats (Morris 1984; Moser et al. 1993, 1995; Moser and Moser 1998). Overall, our findings suggest that water-based cognitive tests may introduce confounding factors in tree shrews, whereas behavioral paradigms in dry-land mazes are more likely to produce optimal results when assessing spatial memory in this species. Our study provides insights into the comparative cognitive abilities of different species and highlights the importance of considering species-specific factors when designing and interpreting cognitive experiments.

### Potential application in spatial cognitive studies

Tree shrews are phylogenetically close to primates (Fan et al. 2013, 2019), reflected not only by similar genome and gene expression pattens (Xu et al. 2012; Fan et al. 2013, 2018), but also by similar brain morphology (Remple et al. 2006, 2007; Wong and Kaas 2009; Wang et al. 2013; Ni et al. 2016, 2018; Dai et al. 2017) and comparable higher-order cognitive functions (Mustafar et al. 2018; Jiang et al. 2021; Pan et al. 2022). In addition to disease models (Cao et al. 2003; Xiao et al. 2017; Yao 2017; Li et al. 2018), the tree shrew also has advantages as an animal model in cognitive studies of spatial navigation (Savier et al. 2021) because of its well-developed visual system (MacEvoy et al. 2009; Lee et al. 2016; Petry and Bickford 2019; Tanabe et al. 2022), prefrontal cortex (Parra et al. 2019), and possibly internal navigation system (Finkelstein et al. 2016). Numerous studies have revealed that the entorhinal-hippocampal circuit represents a cognitive map of the external world (Moser et al. 2015; Rowland et al. 2016). However, it remains unclear how animals use this cognitive map to flexibly navigate to spatial goals (Long and Lu 2022; Nyberg et al. 2022). In addition, both traditional and cutting-edge approaches monitoring neural activities in the brain, including *in vivo* electrophysiology (Dimanico et al. 2021) and 2-photon calcium imaging (Lee et al. 2016; Schumacher et al. 2022), are now being transferred from rodents to tree shrews. Collectively, these lines of evidence raise the possibility of tree shrews as ideal and viable animals for cognitive studies related to flexible navigation with multiple goals and dynamic goal-directed routes. The cheeseboard maze, with its vast array of reward site combinations and route choices, is particularly suitable for flexible spatial tasks.

## Supplementary Material

Tree_shrew_spatial_tasks_supplementary_final_bhad283

## Data Availability

The data that support the findings of this study are available from the corresponding author upon reasonable request.

## CRediT author statement

Cheng-Ji Li (Investigation, Methodology), Yi-Qing Hui (Investigation, Methodology), Rong Zhang (Methodology), Hai-Yang Zhou (Investigation), Xing Cai (Data curation, Writing—review & editing), and Li Lu (Conceptualization, Project administration, Software, Supervision, Validation, Visualization, Writing—original draft, Writing—review & editing)

## Code accessibility

All custom MATLAB scripts used in this study are available upon request.
