# Supplementary material for: A comparison of behavioral paradigms assessing spatial memory in tree shrews
Source: Cereb Cortex. 2023 Aug 26;33(19):10303–21. doi: 10.1093/cercor/bhad283 (PMC11640784; doi:10.1093/cercor/bhad283)
Supplement: Tree_shrew_spatial_tasks_supplementary_final_bhad283 [file tree_shrew_spatial_tasks_supplementary_final_bhad283.docx]

**Supplementary tables and figures for:**

**A comparison of behavioral paradigms assessing spatial memory in tree shrews**

Running title: Spatial memory tests for tree shrews

Cheng-Ji Li^1,2,4^, Yi-Qing Hui^1,4^, Rong Zhang^1,2^, Hai-Yang Zhou^1^, Xing Cai^1,2^, & Li Lu^1,2,3*^

^1^ Key Laboratory of Animal Models and Human Disease Mechanisms of Yunnan Province, Kunming Institute of Zoology, Chinese Academy of Sciences, Kunming, Yunnan 650201, China;

^2^ National Research Facility for Phenotypic & Genetic Analysis of Model Animals (Primate Facility), Kunming Institute of Zoology, Chinese Academy of Sciences, Kunming, Yunnan 650107, China;

^3^ Center for Excellence in Brain Science and Intelligence Technology, Chinese Academy of Sciences, Shanghai 200031, China.

^4^ These authors contributed equally.

^*^ Correspondence: luli@mail.kiz.ac.cn, phone number: (+86-871-65155338)

**Table S1. Remaining sizes of hippocampal subregions in each tree shrew.**

Sizes of hippocampal subregions measured in area of healthy pyramidal/granule cell layer. DG: dentate gyrus; Sub: subiculum.

| Animal | HPC | DG (mm^2^) | CA3 (mm^2^) | CA2 (mm^2^) | CA1 (mm^2^) | Sub (mm^2^) | Total (mm^2^) |
| --- | --- | --- | --- | --- | --- | --- | --- |
| TS094 | Left | 3.33 | 3.66 | 0.91 | 8.73 | 2.17 | 18.79 |
|  | Right | 3.05 | 2.44 | 0.42 | 6.89 | 2.17 | 14.97 |
|  | Sum | 6.38 | 6.10 | 1.33 | 15.62 | 4.34 | 33.76 |
| TS095 | Left | 3.26 | 1.65 | 0.35 | 2.33 | 1.25 | 8.84 |
|  | Right | 2.00 | 0.43 | 0.28 | 1.72 | 0.58 | 5.01 |
|  | Sum | 5.26 | 2.08 | 0.63 | 4.05 | 1.83 | 13.85 |
| TS096 | Left | 7.40 | 8.23 | 0.95 | 6.92 | 5.64 | 29.13 |
|  | Right | 10.60 | 8.88 | 1.14 | 7.47 | 4.60 | 32.69 |
|  | Sum | 18.00 | 17.11 | 2.09 | 14.39 | 10.24 | 61.82 |
| TS097 | Left | 21.00 | 14.50 | 0.79 | 19.09 | 6.91 | 62.29 |
|  | Right | 11.79 | 9.18 | 1.08 | 9.84 | 4.55 | 36.44 |
|  | Sum | 32.79 | 23.68 | 1.87 | 28.93 | 11.46 | 98.73 |
| TS099 | Left | 3.02 | 1.70 | 0.46 | 3.17 | 1.10 | 9.44 |
|  | Right | 3.20 | 1.65 | 0.43 | 7.15 | 2.51 | 14.94 |
|  | Sum | 6.22 | 3.35 | 0.89 | 10.32 | 3.61 | 24.38 |
| TS100 | Left | 26.47 | 14.79 | 1.21 | 18.62 | 9.85 | 70.95 |
|  | Right | 12.83 | 10.49 | 1.14 | 7.96 | 6.09 | 38.52 |
|  | Sum | 39.30 | 25.28 | 2.35 | 26.58 | 15.94 | 109.47 |
| TS101 | Left | 2.39 | 2.34 | 0.32 | 1.85 | 0.70 | 7.59 |
|  | Right | 2.18 | 2.44 | 0.40 | 2.29 | 0.60 | 7.90 |
|  | Sum | 4.57 | 4.78 | 0.72 | 4.14 | 1.30 | 15.49 |
| TS102 | Left | 1.61 | 1.66 | 0.20 | 1.07 | 0.11 | 4.65 |
|  | Right | 1.51 | 1.50 | 0.17 | 1.09 | 0.35 | 4.62 |
|  | Sum | 3.12 | 3.16 | 0.37 | 2.16 | 0.46 | 9.27 |
| TS103 | Left | 12.71 | 10.30 | 1.21 | 9.13 | 6.77 | 40.11 |
|  | Right | 13.59 | 9.34 | 0.97 | 10.22 | 6.83 | 40.94 |
|  | Sum | 26.30 | 19.64 | 2.18 | 19.35 | 13.60 | 81.05 |
| TS106 | Left | 8.25 | 2.27 | 0.13 | 2.05 | 1.87 | 14.57 |
|  | Right | 16.95 | 6.62 | 0.48 | 7.92 | 2.76 | 34.72 |
|  | Sum | 25.20 | 8.89 | 0.61 | 9.97 | 4.63 | 49.29 |
| TS108 | Left | 3.09 | 2.36 | 0.38 | 2.65 | 1.43 | 9.92 |
|  | Right | 12.56 | 8.22 | 0.91 | 7.68 | 4.60 | 33.98 |
|  | Sum | 15.65 | 10.58 | 1.29 | 10.33 | 6.03 | 43.90 |

**Table S2. Remaining sizes of hippocampal subregions correlated with task performance.**

Sizes of hippocampal subregions correlated significantly with residual hippocampal volume (columns 2&3), reference memory averaged across test days in radial-arm maze (columns 4&5), and average route scores in cheeseboard maze (columns 6&7). DG: dentate gyrus.

| Subregion | Hippocampal volume | | Reference memory in radial-arm maze | | Best route score in cheeseboard maze | |
| --- | --- | --- | --- | --- | --- | --- |
|  | r | *P* | r | *P* | r | *P* |
| DG | 0.939 | <0.001 | –0.807 | 0.005 | 0.505 | 0.113 |
| CA3 | 0.989 | <0.001 | –0.754 | 0.012 | 0.605 | 0.049 |
| CA2 | 0.904 | <0.001 | –0.689 | 0.028 | 0.667 | 0.025 |
| CA1 | 0.951 | <0.001 | –0.717 | 0.020 | 0.619 | 0.042 |
| Subiculum | 0.971 | <0.001 | –0.749 | 0.013 | 0.711 | 0.014 |
| Total | 0.996 | <0.001 | –0.780 | 0.008 | 0.613 | 0.045 |


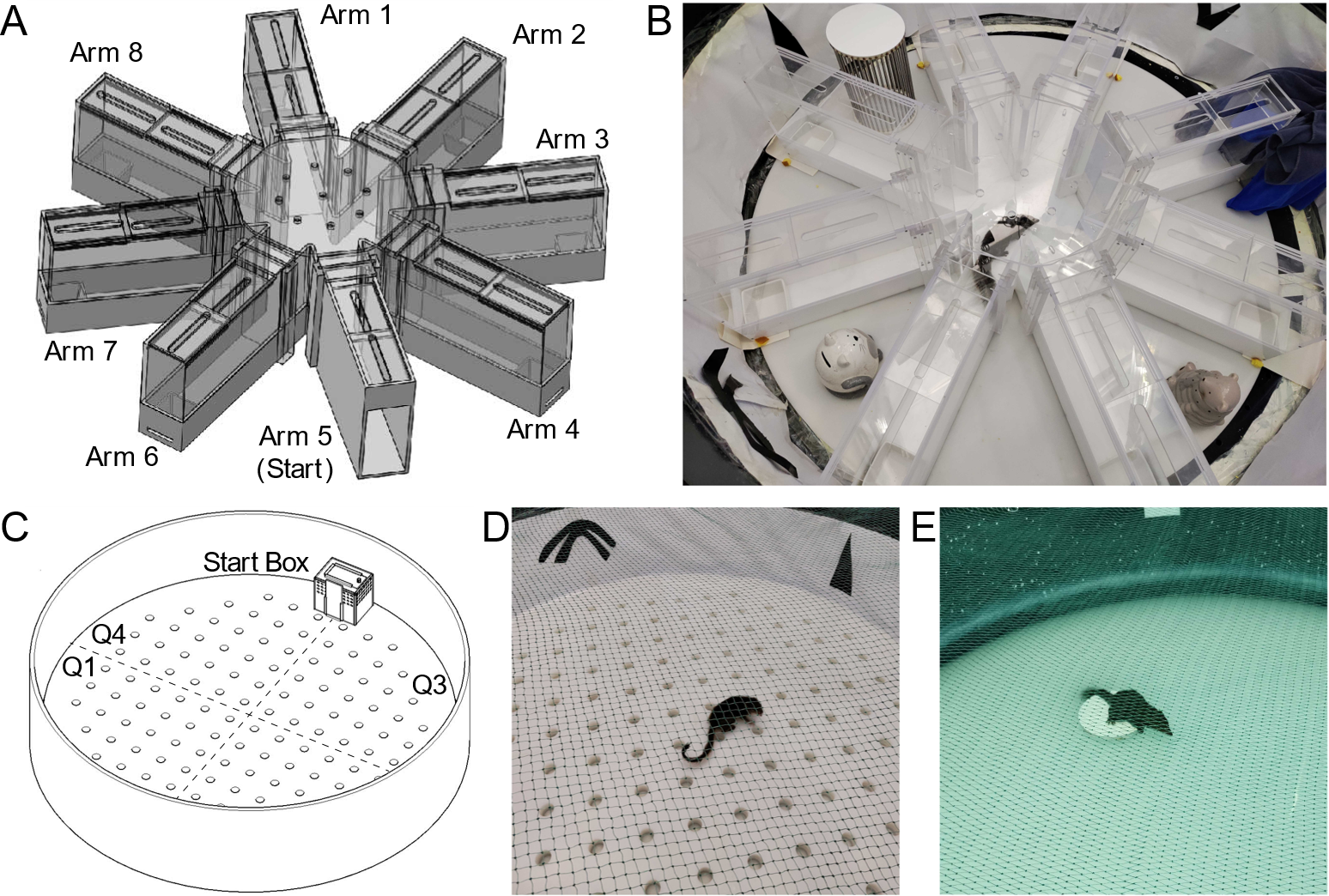


**Fig. S1. Behavior apparatus.**

A) Design of tree shrew radial-arm maze, with transparent walls and lids to facilitate animal’s perception of environmental cues. The start arm (arm 5) contains a large opening that connects to a transfer box. B) Radial-arm maze surrounded by various visual cues. A tree shrew in the maze center is performing the task. C) Schematic of cheeseboard maze. D) Cheeseboard maze decorated with visual cues. A tree shrew in the maze is performing the task. E) A tree shrew seated on the visible platform in water maze pretraining.


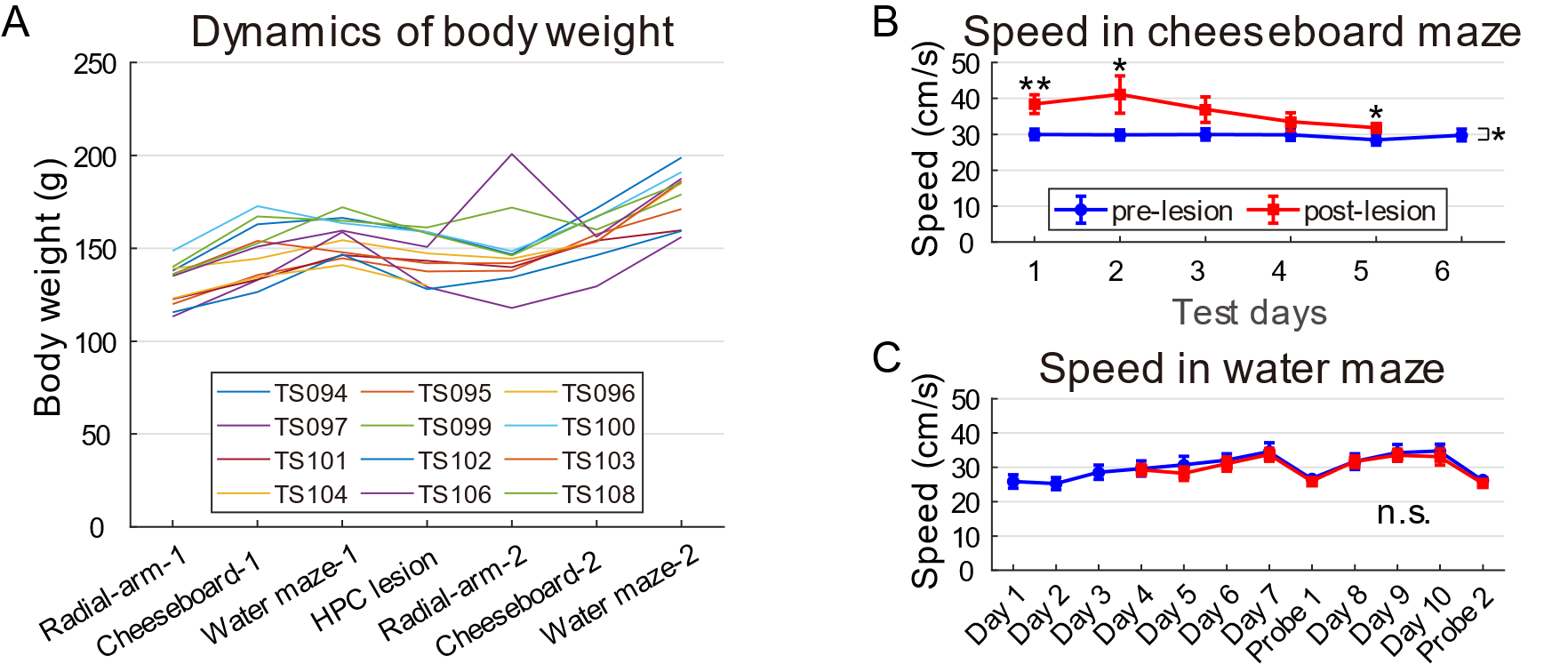


**Fig. S2. Tree shrew body weight and speed in the experiments.**

A) Tree shrew body weight at the beginning of each task/procedure. Colored lines represent data from individual animals. Body weight remained largely constant with two exceptions: TS097 and TS106 had a dramatic decrease and increase, respectively after hippocampal lesions. B) Running speeds (mean±SEM, filtered with a 2.5 cm/s threshold) in cheeseboard maze before and after hippocampal lesions. Tree shrews ran faster after lesion, especially in the first two days (two-way ANOVA for repeated measures, 11 animals, test day: F(4)=4.053, *P*=0.008, η^2^=0.288; lesion: F(1,4)=7.833, *P*=0.019, η^2^=0.439; lesion×test day: F(1,4)=2.699, *P*=0.044, η^2^=0.213). C) Swimming speeds (mean±SEM, filtered with a 2.5 cm/s threshold) in water maze did not differ before and after lesion (two-way ANOVA for repeated measures, test day: F(6)=7.138, *P*<0.001, η^2^=0.417; lesion: F(1)=1.025, *P*=0.335, η^2^=0.093; lesion×test day: F(1,6)=0.269, *P*=0.949, η^2^=0.026). n.s., not significant, *, *P*<0.05, **, *P*<0.01.


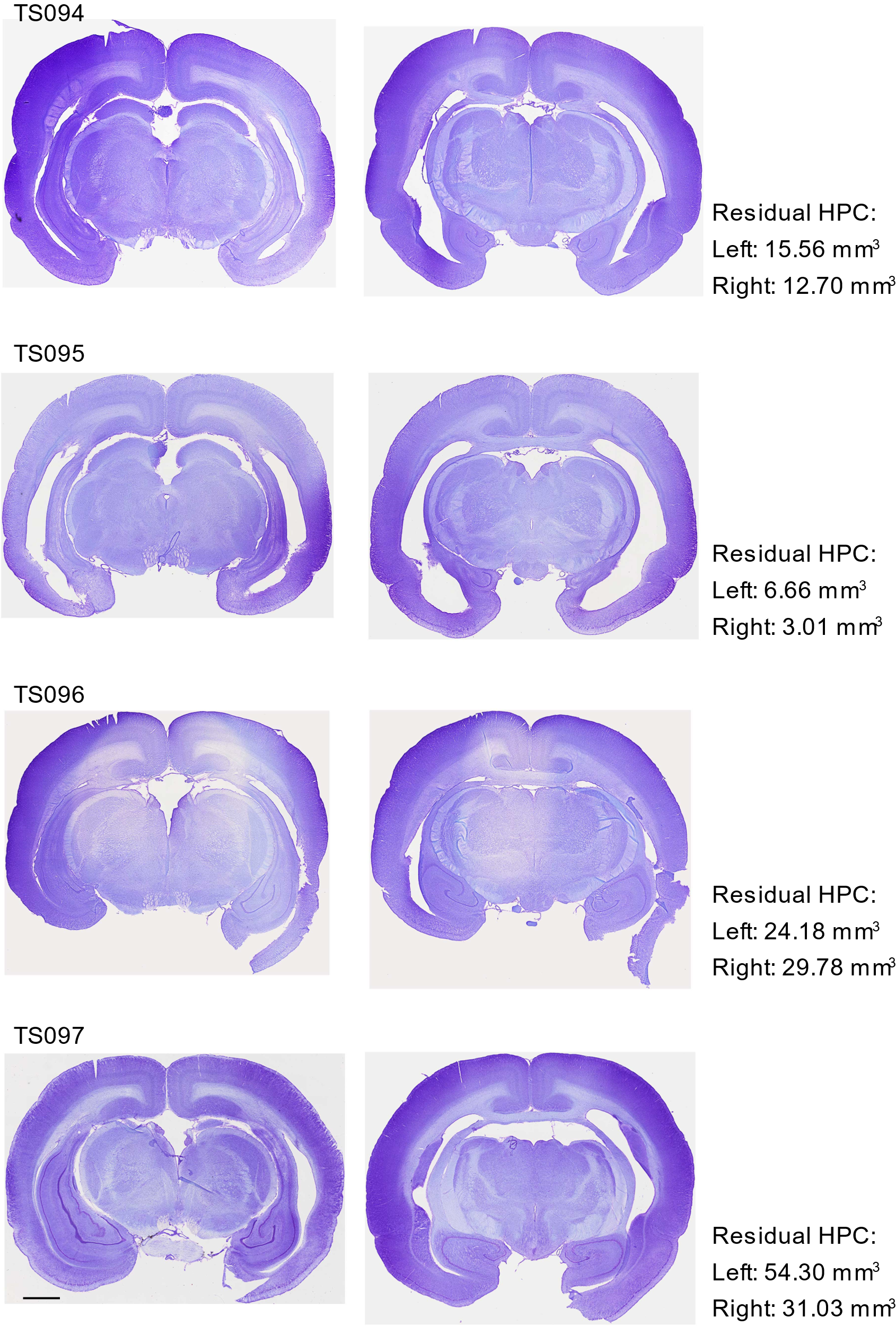


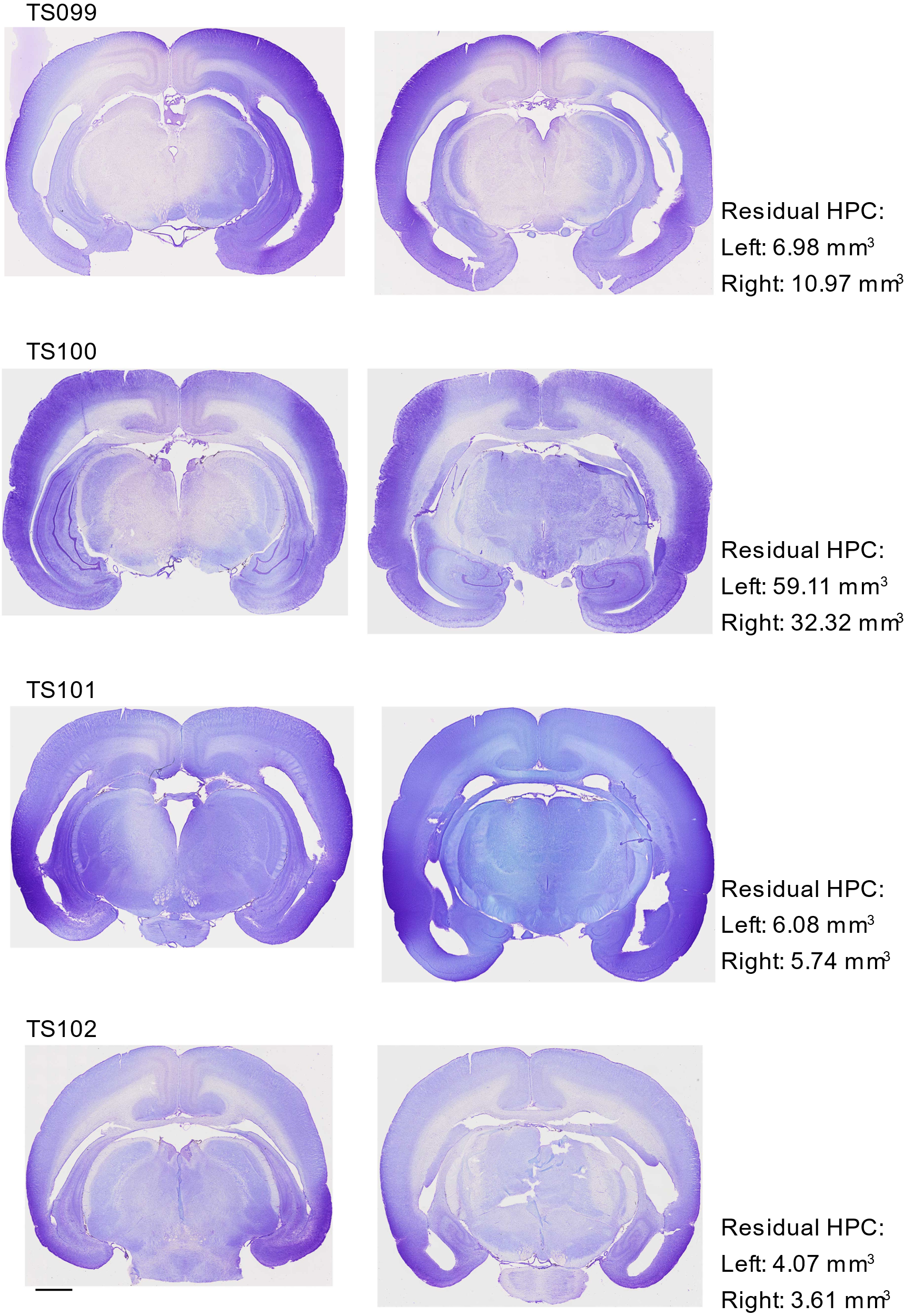


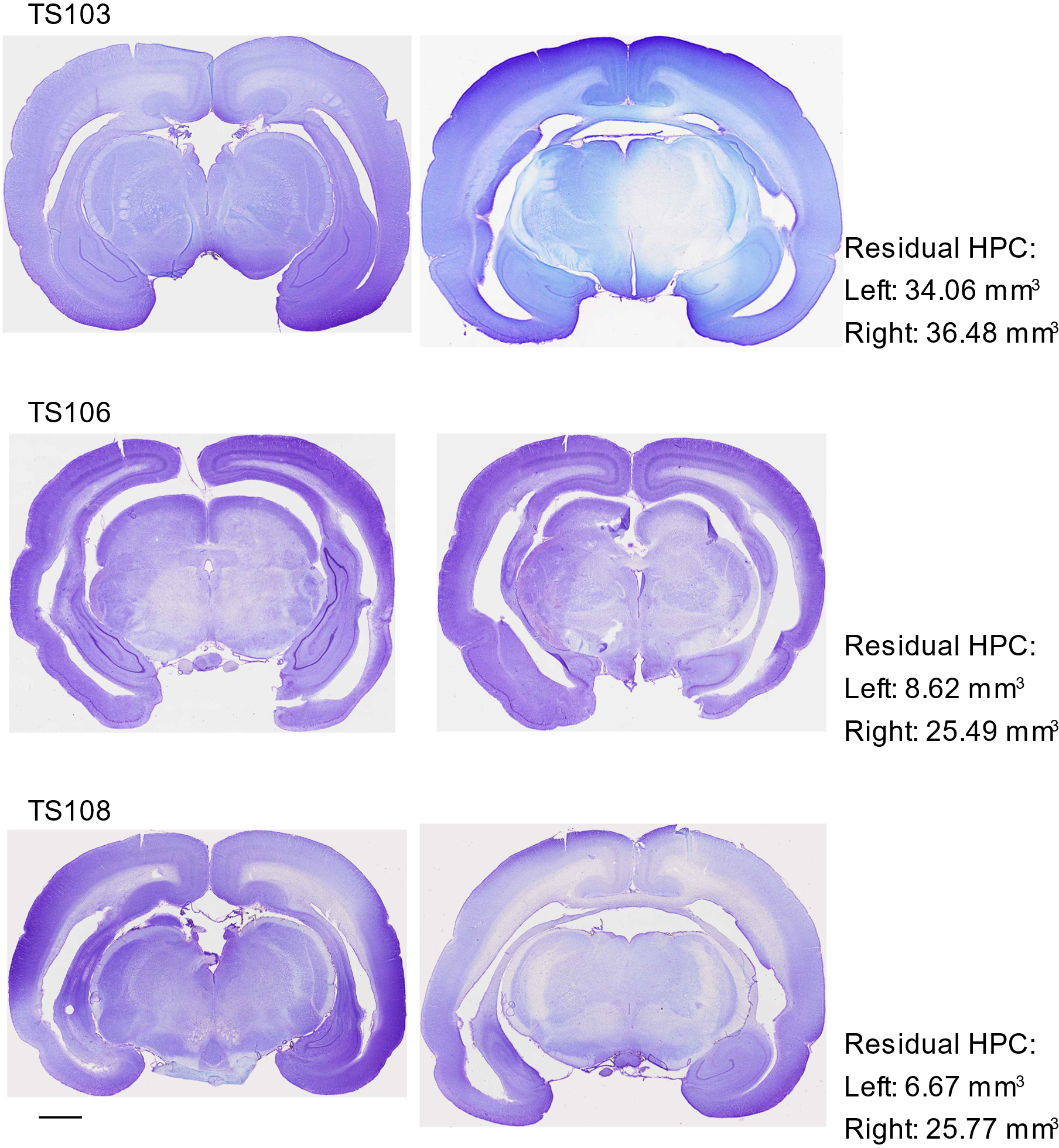


**Fig. S3. Representative histology showing hippocampal lesions in each tree shrew.**

Brain sections of similar levels from each animal are displayed. Volumes of residual hippocampal tissue in each hemisphere are indicated on the right (HPC: hippocampus). Scale bar: 2 mm.


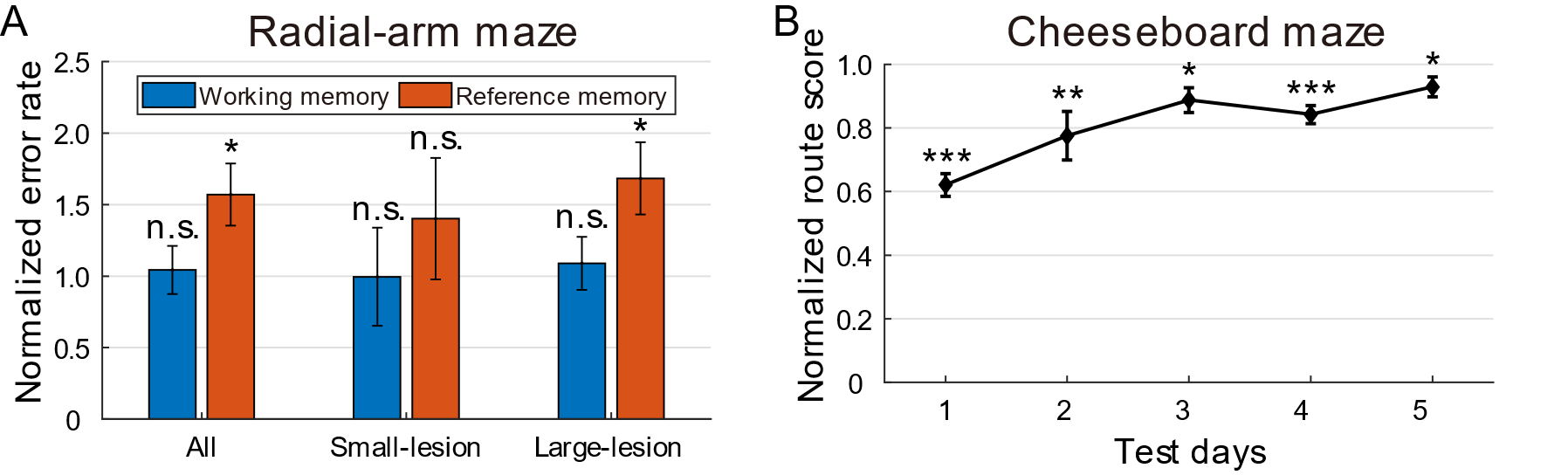


**Fig. S4. Normalized task performance in radial-arm and cheeseboard mazes post lesion.**

A) Normalized error rates (mean±SEM, post-lesion error rates divided by pre-lesion error rates) averaged across test days in radial-arm maze. Normalized reference memory increased significantly after lesion (higher than 1) in tree shrews with large hippocampal lesions, while working memory remained unchanged (one-sample *t*-test, working memory: all animals: t(9)=0.255, *P*=0.804; small-lesion: t(3)=−0.014, *P*=0.989; large-lesion: t(5)=0.479, *P*=0.652; reference memory: all animals: t(9)=2.622, *P*=0.028; small-lesion: t(3)=0.945, *P*=0.415; large-lesion: t(5)=2.712, *P*=0.042). B) Normalized route scores (mean±SEM, post-lesion route score divided by pre-lesion route score) on each test day in cheeseboard maze. Best performance decreased significantly (smaller than 1) on all test days (one-sample *t*-test, test day 1: t(10)=−10.032, *P*<0.001; test day 2: t(10)=−2.810, *P*=0.009; test day 3: t(10)=−2.745, *P*=0.010; test day 4: t(10)=−5.342, *P*<0.001; test day 5: t(10)=−2.150, *P*=0.029). n.s., not significant, *, *P*<0.05, **, *P*<0.01, ***, *P*<0.001.
